# Towards enhancing coral heat tolerance: a “microbiome transplantation” treatment using inoculations of homogenized coral tissues

**DOI:** 10.1186/s40168-021-01053-6

**Published:** 2021-05-06

**Authors:** Talisa Doering, Marlene Wall, Lalita Putchim, Tipwimon Rattanawongwan, Roman Schroeder, Ute Hentschel, Anna Roik

**Affiliations:** 1 GEOMAR, Helmholtz Centre for Ocean Research, Kiel, Germany; 2 Phuket Marine Biological Center (PMBC), Phuket, Thailand; 3 Christian-Albrechts University of Kiel, Kiel, Germany

**Keywords:** Microbiome transplantation, Marine microbiomes, Climate change, Microbiome flexibility, Thermal tolerance, Beneficial bacteria, 16S rRNA gene, Coral bleaching, Assisted evolution

## Abstract

**Background:**

Microbiome manipulation could enhance heat tolerance and help corals survive the pressures of ocean warming. We conducted coral microbiome transplantation (CMT) experiments using the reef-building corals, *Pocillopora* and *Porites*, and investigated whether this technique can benefit coral heat resistance while modifying the bacterial microbiome. Initially, heat-tolerant donors were identified in the wild. We then used fresh homogenates made from coral donor tissues to inoculate conspecific, heat-susceptible recipients and documented their bleaching responses and microbiomes by 16S rRNA gene metabarcoding.

**Results:**

Recipients of both coral species bleached at lower rates compared to the control group when exposed to short-term heat stress (34 °C). One hundred twelve (*Pocillopora* sp.) and sixteen (*Porites* sp.) donor-specific bacterial species were identified in the microbiomes of recipients indicating transmission of bacteria. The amplicon sequence variants of the majority of these transmitted bacteria belonged to known, putatively symbiotic bacterial taxa of corals and were linked to the observed beneficial effect on the coral stress response. Microbiome dynamics in our experiments support the notion that microbiome community evenness and dominance of one or few bacterial species, rather than host-species identity, were drivers for microbiome stability in a holobiont context.

**Conclusions:**

Our results suggest that coral recipients likely favor the uptake of putative bacterial symbionts, recommending to include these taxonomic groups in future coral probiotics screening efforts. Our study suggests a scenario where these donor-specific bacterial symbionts might have been more efficient in supporting the recipients to resist heat stress compared to the native symbionts present in the control group. These findings urgently call for further experimental investigation of the mechanisms of action underlying the beneficial effect of CMT and for field-based long-term studies testing the persistence of the effect.

Video abstract

**Supplementary Information:**

The online version contains supplementary material available at 10.1186/s40168-021-01053-6.

## Introduction

Reef-building corals are subjected to heat stress due to ocean warming. Frequent heatwaves induce coral bleaching, the disruption of the symbiosis between the coral host and its dinoflagellate symbionts. With ongoing ocean warming, coral bleaching events that entail high mortality have increased over the last decades and are expected to intensify [1], which calls for interventions that can enhance coral resilience. One such concept is “assisted evolution,” encompassing selective breeding of corals and the manipulation of coral-associated microbiome communities, like dinoflagellate symbionts and bacteria [2]. Multi-generational coral breeding studies are aiming to select for heat-tolerant offspring, but will require larger time scales [3]. However, manipulation of the fast-evolving microbiome could have substantial effects on much shorter time scales [4, 5]. Corals are associated with a diversity of microbes, such as dinoflagellates, other protists, fungi, bacteria, archaea, and viruses [6]. While we know that coral holobiont functioning relies on the supply of fixed carbon and several essential amino acids by dinoflagellate symbionts [7, 8], the roles of other holobiont members are still widely elusive [9]. Among all those other holobiont members, coral-associated bacterial communities (in the following “the microbiome”) have been studied and characterized for the past two decades [10–14] and we learned that they likely support diverse metabolic processes of the holobiont [8, 15–17]. Additionally, the coral microbiome can benefit the host through provision of vitamins, antioxidants, and antimicrobials, hence protecting against stressors and pathogens [8, 18]. Microbiome communities are coral species-specific, but can differ across space, time, and respond to environmental drivers, while certain parts of the microbiome are suggested to constitute a stable “core” community [6, 13, 14, 19–22]. Presumably, the flexibility of microbiome communities should allow manipulation by, e.g., administration of probiotics with the goal of promoting holobiont resilience [4]. Probiotic treatments have already proven to be an effective tool to tweak host health and performance in agriculture, insect model organisms, and human medicine. For instance, inoculations of crop plants with beneficial bacterial consortia have been performed to increase crop yields or to ward off plant pathogens [23]. Heat tolerance of the pea aphid, an insect model organism, was successfully enhanced through inoculation with a heat-tolerant strain of its obligate bacterial symbiont [24]. Particularly, approaches of human gut microbiome “transplantation” (i.e., fecal microbiome transplantations a.k.a. FMTs) have emerged as successful therapies relying on transmission of living, beneficial microbiomes from a healthy human donor to a symptomatic patient. Most prominently, FMTs are being employed as a treatment for several gastrointestinal conditions [25].

Microbiome manipulation for corals is still is in its infancy. Nonetheless, pioneering studies have demonstrated feasibility by showing that microbiome communities can be shaped through inoculation with cultured bacterial isolates or phages [26–29], while several have already taken the first steps of testing the probiotic potential of these inoculations, i.e., monitoring whether manipulation treatments are accompanied with improvements of coral health and resistance particularly under heat, pathogen, and pollutant stress [26, 30–32]. To further advance coral microbiome manipulation techniques, we set out to assess the effects of a field-based coral microbiome transplantation (CMT) procedure which intends to inoculate heat-sensitive corals with donor microbiomes using fresh tissue homogenates produced from heat-tolerant conspecific donor corals. This CMT strategy bypasses time-consuming culturing and screening for beneficial bacteria from healthy donors and importantly enables the transmission of the “unculturable” microbiome fraction. A cautious selection of healthy donors is crucial in order to minimize the undesired transmission of pathogens or pollutant agents during the procedure. We tested CMT for two cosmopolitan reef-building corals, *Pocillopora* sp. and *Porites* sp., from the Andaman Sea in Thailand. First, we assessed heat stress tolerance by employing short-term heat stress assays (*sensu* Oliver and Palumbi et al. [33] and Voolstra et al. [34]) in order to identify suitable donors and recipients in wild coral populations. We focused on high variability habitats that likely host corals of higher heat stress tolerance, while hypothesizing that corals from sheltered reefs of near-optimal reef conditions would display heat stress sensitivity [35, 36]. 16S rRNA gene metabarcoding was performed throughout the CMT procedure teaching us new lessons of bacterial uptake and microbiome flexibility. Most importantly, the reassessment of heat tolerance after inoculation indicated a beneficial effect of the CMT treatment. These results call for investigations to further explore its underlying mechanism of action of the observed beneficial effect and whether the CMT method has the potential to be developed towards a feasible probiotic intervention supporting coral health during heatwave events.

## Results

**Fig. 1:**
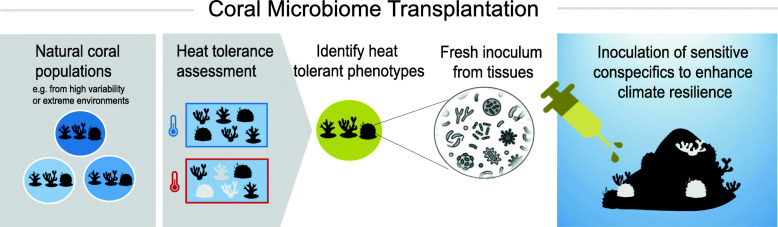
Coral microbiome transplantation. A field-based probiotic strategy to support coral heat tolerance during global ocean warming. Over the past years, microbiome transplantation has already been successfully employed as a clinical therapy for the treatment of several human gastrointestinal disorders. Applied to corals, this technique aims to expose heat-sensitive corals to bacterial communities of heat-resilient conspecific donor corals. Such donors can likely be found in reefs of environmental variability or extremes. CMT has several benefits. It bypasses time-consuming culturing and screening effort of bacterial isolates, which is required for the production of lab-cultured probiotics. Most importantly, this strategy enables the transmission of the “unculturable” fraction of the microbiome. Furthermore, reintroduction of CMT recipient corals will not clash with ethical considerations, since donor colonies can be locally sourced from reef habitats at the location of the application. As such, this approach can become a feasible, local management strategy for coral reefs. As ocean warming is progressing rapidly, an expeditious strategy like the CMT could represent a powerful probiotic intervention for corals

We present the results from two coral microbiome transplantation experiments with the underlying concept of using a fresh tissue homogenate of heat-tolerant donors from the wild, containing living microbiome communities, for the inoculation of heat-sensitive conspecifics to enhance their resilience under ocean warming (Fig. 1).

### Heat tolerance assessment of corals in the wild

**Fig. 2:**
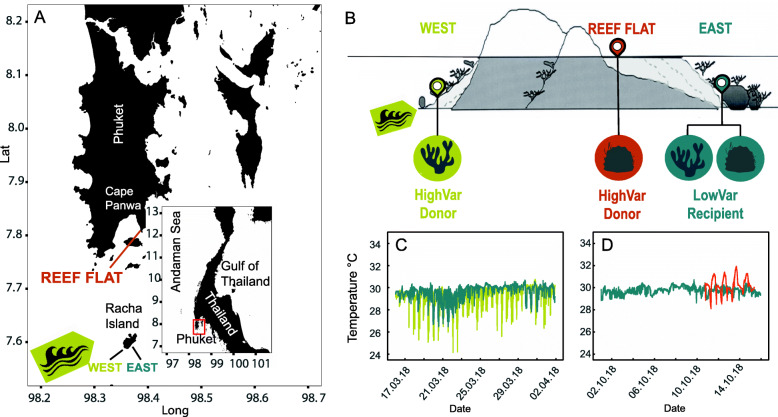
Collection sites and environmental properties. **a** Coral sites at Racha Island and Panwa reef flat in the Andaman Sea south of Phuket Island in Thailand (red rectangle). The impact of large-amplitude internal waves creates high variability habitats on the western shore of Racha Island (light green arrow). At the shallow reef flat in Panwa, high variability and extreme conditions are linked to diurnal solar and tidal variation. **b** Three distinct reef sites were selected: a high variability west shore site of Racha Island (“HighVar,” 15 m depth, light green); a high variability reef flat in Phuket Island, Panwa (“HighVar,” 0–2 m depth, orange); and a sheltered low variability reef site of stable environmental conditions at Racha Island east shore (“LowVar,” 15 m, teal). Corals from “HighVar” environments were designated as microbiome donors, whereas corals from the “LowVar” site were used as recipients during microbiome transplantation experiments. **c**, **d** In situ temperature profiles show the temperature history of corals prior to experiments. Strong fluctuations of temperature were measured at the “HighVar” west shore site (light green in **c**) and reef flat (orange in **d**), while comparably stable conditions are shown for the “LowVar” east shore site (teal in **c** and **d**). Branching coral, *Pocillopora* sp.; massive coral, *Porites* sp.

**Fig. 3:**
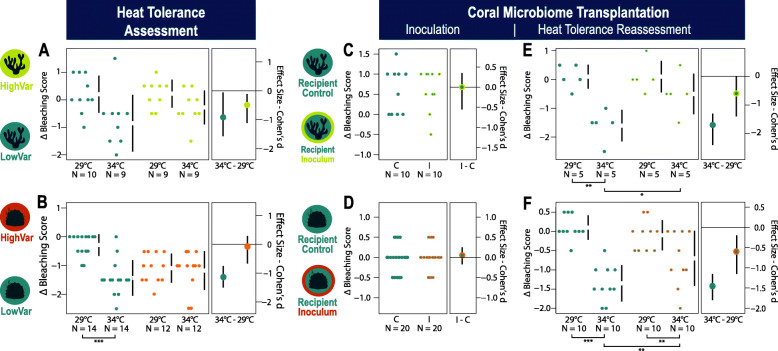
Coral bleaching responses during heat tolerance assessments before and after the coral microbiome transplantation experiments. **a**, **b** Effects of temperature (“29 °C” vs. “34 °C”) on the bleaching score of corals from sites of low and high environmental variability (“LowVar” and “HighVar”) are compared. **c**, **d** Next, data shows that the handling procedure during inoculation had no impact on the bleaching score of corals (“I”, inoculation group vs. “C”, sterile-filtered seawater (FSW) control group). Subsequently, **e**, **f** the temperature effects on the bleaching scores of the recipient group and the FSW control group are shown. Plots visualize ∆-bleaching score data (i.e., the difference of tissue color intensity at end–start of each experimental part). Swarm plots (left side plot) show raw data points and Cumming estimation plots (right) depict the effect sizes as the mean differences between the treatment groups using Cohen’s *d* and a 95% confidence interval. Significant differences are indicated by connecting lines (*p* < 0.001***, < 0.01**, < 0.05* from generalized linear/linear mixed effect models). Vertical error bars, 95% CI; *N*, individuals per treatment group; branching coral, *Pocillopora*; massive coral, *Porites*; light green, “HighVar” west shore corals; orange, “HighVar” reef flat corals; teal, “LowVar” east shore corals; colored circles represent the donor inoculum used: light green, “HighVar” *Pocillopora* donor; orange, “HighVar” *Porites* donor

First, to identify suitable donor corals, we assessed heat stress responses of two coral species, *Pocillopora* and *Porites*, by screening fragments from wild colonies living in high and low variability reef sites, “LowVar” and “HighVar,” in the Andaman Sea (Fig. 2a-d). We used short-term heat stress assays (Fig. S1 A-B) and measured coral response variables such as the bleaching score of the coral tissues and photosynthetic efficiency of dinoflagellate symbionts to gain insight into stress condition before and after the heat exposure (Dataset S1). Corals of both species originating from the “HighVar” sites had a higher bleaching resistance compared to their “LowVar” conspecifics, as indicated by the overall decreased ∆-bleaching score of “LowVar” fragments under heat exposure (effect sizes ~ −1.0 and −1.1, Fig. 3a, b). In contrast, no significant changes were documented for “HighVar” corals under elevated temperature in the heat stress assays (effects sizes ~ −0.1 and −0.3). In our assays, this differential heat tolerance, “LowVar” vs. “HighVar” was slightly more pronounced for *Porites* than for *Pocillopora. Porites* from the “LowVar” site bleached significantly (*p* < 0.001), whereas their “HighVar” conspecifics exhibited no significant response under heat exposure (Tables S1-2). In contrast, the effect of heat exposure on pocilloporid corals from the “LowVar” site marginally failed to be statistically significant (effect size ~ −1.0, *p* = 0.051). Yet, the effect of heat exposure on “LowVar” corals was larger compared to the minor effect on “HighVar” conspecifics (effect size ~ −0.4), which hardly showed a bleaching score decline under heat exposure. Furthermore, only *Pocillopora* fragments showed a differential response between the two sites of origin based on photosynthetic efficiency (Fig. S2 A-B). Here, photosynthetic efficiency decreased in “LowVar” corals under heat exposure (*p* = 0.045, Tables S1-2), while it did not change for “HighVar” corals. Photosynthetic efficiency of *Porites* fragments from both sites significantly decreased under heat exposure (*p* = 0.001), showing no difference in the stress response of the dinoflagellate symbionts. Based on these outcomes, corals from the “LowVar” site were designated to be recipients, while the more heat-tolerant “HighVar” corals were used as microbiome donors in the CMT experiments.

### Heat tolerance assessment of recipients after inoculation

The two CMT experiments, one for each coral species, consisted of two parts respectively (Fig. S1 C-D): first, the inoculation procedure, in which the treatment group received a donor-inoculum (“I”: recipients) and a control group received an inoculation treatment of sterile-filtered seawater (“C”: FSW control group); second, the reassessment of heat tolerance, where both groups were exposed to either a heat treatment of 34 °C or ambient of 29 °C resulting in four treatment groups, i.e., “I x 29 °C,” “I x 34 °C,” “C x 29 °C,” and “C x 34 °C.” The mere inoculation procedure did not entail any changes in the bleaching score and the photosynthetic efficiency (Fig. 3c,d and S2 C-D, Tables S1-2). However, it subsequently had a beneficial effect on the bleaching resistance of recipients when exposed to heat, as the pocilloporid “I” recipients did not significantly bleach under heat exposure (effect size ~ −0.5), but corals in the FSW control group bleached measurably (effect size ~ −1.6, *p* = 0.002, Fig. 3e). *Porites* “I” recipients were not entirely immune to bleaching, as the effect of heat exposure was significant and the bleaching score slightly reduced (effect size ~ −0.6, *p* = 0.004, Fig. 3f). Yet, in comparison, the stress response of the FSW control group “C” was far more severe (effect size ~ −1.5, *p* < 0.001), suggesting a beneficial effect of CMT on *Porites* recipients.

Measurements of photosynthetic efficiency did not reflect the beneficial effect of CMT in the same manner as shown by the bleaching score (Fig. S2 E-F). Over the course of time, the heat exposure led to a significant decrease of photosynthetic efficiency in both groups, “I x 34 °C” and “C x 34 °C.” However, at the end of the heat stress assays, there was no significant difference in photosynthetic efficiency levels between these two groups (Tables S1-2).

### Microbiome data overview

To sequence DNA for microbiome analysis, coral and seawater samples were collected at three time points during the two CMT experiments, at the “start” and “end” of inoculation and at the “end” of heat tolerance reassessment (Fig. S1 C-D). α- and β-diversity analyses of the amplicon data rely on a rarefied data set containing 7177 amplicon sequence variants (ASVs) over 210 samples with 4000 reads per sample (Fig. S3). Further analyses used a filtered data set (i.e., “filt-10” data), where low abundant ASVs were removed from the original data resulting in 4604 ASVs over 293 samples and 2,335,885 reads in total (more details in *Supplementary Materials and Methods*; Dataset S2). Overall, the samples clustered by experiment, reflecting two different microbiome communities of the two coral species (*p*_permanova_ < 0.001). Source seawater tank communities were remarkably distant to both coral microbiomes, whereas seawater communities of experimental tanks were closer to the respective coral microbiomes (Fig. S4). This suggests that coral microbiome communities influence their immediate microbial surrounding to a certain degree rather than the opposite. Corals contained a high number of unique ASVs (~1500–2300) which made up 42% and 65% of all coral sequence reads in *Pocillopora* sp. and *Porites* sp., respectively. Comparably, very few ASVs (~70–400) were shared with the seawater microbiomes (Fig. S5).

### Coral microbiome compositions

Pocilloporid microbiomes were mainly composed of Proteobacteria, Bacteroidetes, and a smaller proportion of Chloroflexi and Actinobacteria. Additionally, donors and inoculum were associated with Cyanobacteria and an unclassified taxon. Within these microbiomes, three bacterial species were strongly dominant, i.e., *Candidatus Amoebophilus* sp. (up to 90%), *Alteromonas* sp. (up to 62%), and a species of Rhodobacteraceae (up to 40%) (Fig. S6 A). Proteobacteria prevailed in the *Porites* microbiomes and some individuals had a large proportion of Tenericutes, Bacteroidetes, and Epsilonbacteraeota. *Endozoicomonas* sp. was the solely dominant bacterial species associated with *Porites* corals (up to 99%). In few individuals, *Alteromonas* sp. and a species of Entomoplasmatales were prevalent, but comparably less abundant (Fig. S6 B).

### Microbiome differences between the donors and recipients at the experiment start

Both CMT experiments started with rather similar α-diversity metrics of the donor, inoculum, and recipient microbiomes (Fig. S7). Only *Porites* recipients initially had a slightly higher richness and significantly lower evenness compared to the donor and inoculum microbiomes (*p* = 0.058 and 0.045, respectively, Table S3), due to the dominance of *Endozoicomonas*. However, several compositional differences (β-diversity) were significant between the three sample groups at the experiment start, i.e., inoculum, donor samples from field collection, and recipient samples (*p*_permanova_ = 0.001 for both coral species, Fig. S8). Most notably, the β-diversity distance between the “donor+inoculum” microbiome community to the recipients’ community was particularly large in *Porites* (both *p*_pairwise permanova_ < 0.01, Tables S4-5) and marginal in *Pocillopora*, where the “donor+inoculum” community was comparably less distant from the recipients.

When focusing on the microbiomes at the experiment start (based on “filt-10” data), we find that 146 ASVs were exclusively found in the pocilloporid inoculum, which represent potentially new donor bacteria, not yet present in the recipients’ microbiome prior to CMT (Fig. S9 A). Thirty ASVs were exclusively found in the *Porites*-donor inoculum (Fig. S9 B) when compared to the sequenced entirety of the recipients’ microbiomes. Interestingly, in *Pocillopora*, five *Peredibacter*-like species (phylogenetic order: Bdellovibrionales) and one *Halobacteriovorax* sp. were most abundant among these exclusive inoculum-specific ASVs, contributing to ~3% of the entire sequenced inoculum bacterial community. Other ASVs (0.1–0.3%) were an α-proteobacterium (Dstr-E11), three unclassified bacteria (uncultured Mollicutes), six Rhodobacteraceae, and five Alteromonadaceae. In *Porites*, six species of *Endozoicomonas* sp. were most prominent contributing to 3% of the entire inoculum community. To be considered, the “filt-10” data set represents the pocilloporid inoculum with 904,749 reads and *Porites* inoculum with only half of that (Fig. S9), which could be related to a sequencing bias or reflect a biological characteristic.

### Bacterial densities during inoculation

Inoculations were performed in semi-enclosed micro-environments (Fig. S10), where bacterial densities were enriched by addition of the respective inoculum made of homogenized donor coral tissues. Bacterial cell counts showed that pocilloporid recipients were incubated in a bacterial density of 3.4 × 10^5^ cells ml^−1^ inside the enclosures and with 1.5 × 10^4^ cells ml^−1^ once the tubes were removed. *Porites* recipients were exposed to bacterial densities of 1.9 × 10^6^ (day 1), 1.8 × 10^6^ (day 2), and 1.2 × 10^6^ (day 3) cells ml^−1^ inside the tubes and 1.7 × 10^5^, 1.6 × 10^5^, and 1.1 × 10^5^ cells ml^−1^, respectively, when tubes were removed. Due to logistic limitations in the field, we could not perform a dosage test ahead of the experiments. To further develop the CMT method, more testing in this regard should be considered. Follow-up experiments are recommended to adjust bacterial cell densities of inocula prior to inoculation, which will require longer experimental time frames, laboratory space, and coral tissue material, but will allow for measuring dosage-dependent effects.

### Microbiome community changes observed after inoculation

**Fig. 4:**
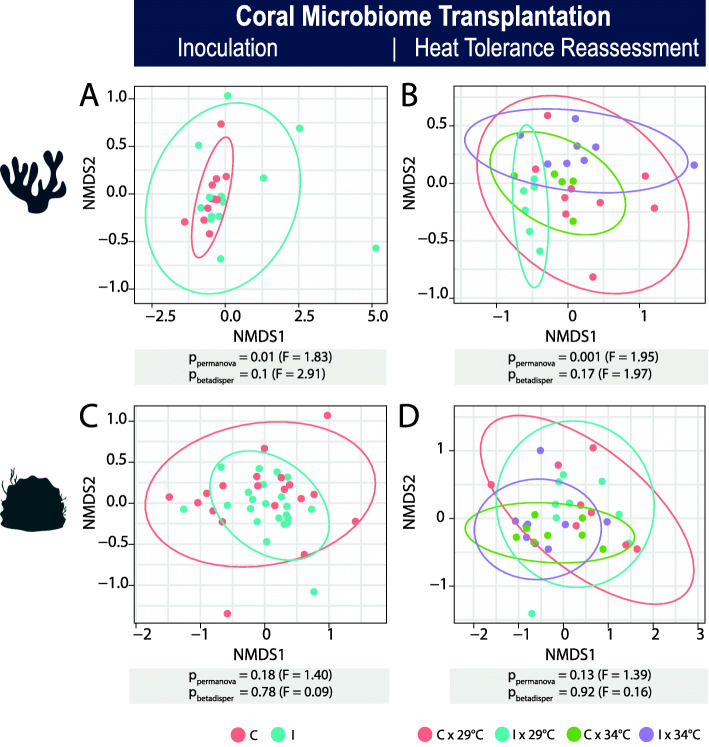
β-diversity of the coral microbiome communities throughout the coral microbiome transplantation experiments. The effects of the inoculation procedure (“I” vs. “C”) and the effects of subsequent heat exposure (“29 °C” vs. “34 °C”) on microbiome communities of “I” recipients and the “C” (FSW sterile-filtered seawater) control group are shown for **a**, **b** *Pocillopora* sp. and **c**, **d** *Porites* sp. Non-metric Multidimensional Scaling (nMDS) plots show microbiome communities based on Bray-Curtis dissimilarities. Group differences based on dissimilarities and dispersion were tested using PERMANOVA and BETADISPER analysis and *p* and *F* values are reported

Significant reshaping of the recipients’ microbiome communities in response to CMT inoculation was demonstrated for *Pocillopora*, but not for *Porites* (Fig. 4). Microbiome diversity was slightly lower for the pocilloporid “I” recipients (Fig. S7, Fig. S11 B), and community structure was significantly changed after inoculation in comparison to the FSW control group (*p*_permanova_ = 0.01, Fig. 4a, Table S4). Subsequent heat exposure did not further affect α-diversity, but the microbiome community was changed in “I” recipients (*p*_permanova_ < 0.001, Fig. 4b). Notably, the differences between the “I” recipients and the FSW control group persisted after the heat stress assay (all *p*_pairwise permanova_ < 0.05, Fig. 4b). In contrast, the microbiomes of *Porites* “I” recipients did not undergo any significant changes measured by α- and β-diversity in response to CMT (Fig. 4c, Fig. S7, Tables S 4-5). Even after heat exposure, *Porites* microbiomes of both groups remained without significant community rearrangement (Fig. 4d). Notably, community richness and diversity increased in both *Porites* groups “I” and “C” under heat exposure, but this effect was not significant (Fig. S7 D-F, Fig. S10 J-L, Table S3).

### Potentially transmitted bacterial species between the inoculum and recipients

**Fig. 5:**
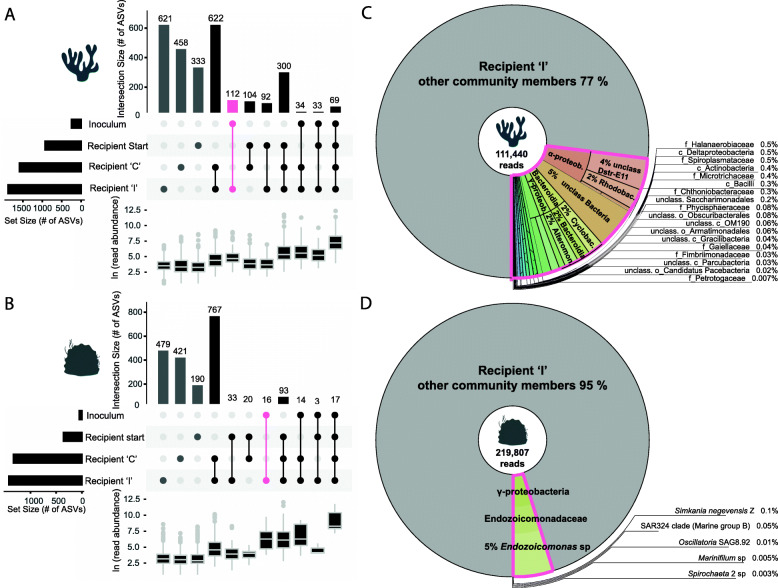
Transmission of bacteria in the coral microbiome transplantation experiments. *UpSetR* analyses identified amplicon sequence variants (ASVs) representing bacteria that were potentially transmitted from the inoculum to the recipient corals during the CMT treatment in **a** *Pocillopora* sp. and **b** *Porites* sp. The transmitted bacterial community, consisting of donor-specific bacteria, is represented by the exclusive overlap between the “inoculum” and “I” recipient microbiomes (marked pink). Set sizes are indicated by horizontal bars and unique and intersection group sizes are indicated by vertical bars. The inoculum set had no unique ASVs. A boxplot depicts the ln-transformed read counts per set, as an indicator of abundance. Krona plots for **c** *Pocillopora* sp. and **d** *Porites* sp. highlight the transmitted bacterial community within the total microbiomes of “I” recipients, showing relative abundances and phylogenetic classification at order, family, and species levels (SILVA database bootstrap > 80)

We identified 112 ASVs as exclusively shared by the microbiomes of the pocilloporid inoculum and the “I” recipient group, thus suggesting these bacterial species were potentially transferred by the CMT procedure (Fig. 5a). Sixteen of such ASVs were identified in the *Porites* experiment (Fig. 5b). The proportion of these donor-specific bacteria in “I” recipients was larger in *Pocillopora* compared to *Porites* (relative abundance of 23% vs. 5%, respectively; Fig. 5c, d). We found members of the Dstr-E11 group, unclassified Mollicutes, Rhodobacterales, *Alteromonas* sp. and other Alteromonadales, and Candidatus *Amoebophilus* sp. and other Cytophagales, as well as Bdellovibrionales occurring at lower abundances among the transmitted ASVs in *Pocillopora* (Table 1, Dataset S3). In *Porites*, donor-specific *Endozoicomonas* sp. ASVs constituted the majority of the transmitted bacterial community.

**Table 1:** Transmission of bacteria in the coral microbiome transplantation experiments. A subset of the 112 transmitted bacterial candidates is shown for *Pocillopora* sp. This subset consists of amplicon sequence variants (ASVs) within the most abundant bacterial orders that contain >200 reads. For *Porites* sp., all 16 transmitted bacterial variants are shown. Bacterial orders [and families] are listed together with the number of ASVs within these taxonomic groups and their relative abundances within the “I” recipient microbiome community. For each bacterial order, up to three most abundant ASVs are listed with their lowest taxonomic classification (SILVA v 132) and nearest relative information from GenBank (NCBI)

***Pocillopora*** **“I” recipient microbiomes**			
**Order [families]**	**Relative abundance**	**No. of ASVs**	**Lowest taxonomic classification (SILVA) | sources and nearest relatives [% similarity; GenBank accession#]**
Dstr-E11 [unclassified]	3.73	1	Unclassified Dstr-E11 | *Montastraea franksi* (coral) [99.75%; GU118824.1], *Diploria strigosa* (coral) [99.75%; GU118205.1]
Bacteria [unclassified] Rhodobacterales [Rhodobacteraceae]	3.57	1	Unclassified Bacterium | Mollicutes bacterium, ascidians in eel pond [85.55%; EF137401.2], *Anemonia viridis* [86.43%; KC862086.1]
	2.43	6	Unclassified Rhodobacteraceae | *Lophelia pertusa* (cold water coral) [97.52%; FJ041447.1], *Stylophora pistillata* (coral) [96.26%; KC669141.1]
			Unclassified Rhodobacteraceae | *Tropicbacter* sp. from marine sponge [100%; KP412857.1, MH818487.1]
			Unclassified Rhodobacteraceae | *Litopenaeus vannamei* gut (shrimp) [99.5%; MK589151.1], diseased *Montastraea faveolata* (coral) [99.5%; FJ203299.1]
Clostridiales [Lachnospiraceae, Ruminococcaceae]	2.11	13	Unclassified Family XIII | endolithic bacteria from marine intertidal outcrop [99.51%; KT977254.1], healthy *Montastraea faveolata* (coral) [97.79%; FJ203646.1]
			*Anaerococcus* sp. | Mouse cecum [100%; KY672406.1]
			*Ruminococcus* torques group | Wastewater treatment system [99.75%; LR637623.1]
Cytophagales [Cyclobacteriaceae, Spirosomaceae, Amoebophilaceae]	1.96	9	Unclassified Cyclobacteriaceae | *Montastraea faveolata*, *Montastraea franksi* (corals) [100%; GU118859.1, GU118562.1]
			*Reichenbachiella* sp. | Dinoflagellate culture [96.93%; KJ754644.1], Gulf of Mexico oil surface [95.27%; KF786696.1]
			Candidatus *Amoebophilus* sp. | *Astrangia poculata*, *Cladocora caespitosa*, *Galaxea fascicularis* (corals) [95.52-95.70%; MK175907.1, KU354136.1, JQ235892.1]
Alteromonadales [Alteromonadaceae]	1.63	8	*Alteromonas* sp. |*Favia* sp. (coral mucus layer adjacent to black band mat) [100%; EF433118.1]
			Two unclassified Alteromonadaceae | *Alteromonas* sp. from Mariana Trench [99.77%; MH010314.1], *Xestospongia muta* (sponge) [99.77%; CP031010.1], Polyhydroxyalkanoates producing bacteria [99.77%; KU521388.1]
Bacteria [unclassified]	1.21	1	Unclassified bacteria | Mollicutes bacterium from ascidians in eel pond [85.32%; EF137401.2], *Anemonia viridis* [86.18%; KC862086.1]
Flavobacteriales [Flavobacteriaceae, Weeksellaceae]	0.74	7	*Tenacibaculum* sp. | *Flexibacter aurantiacus* subsp. Copepodarum [99.06%; AB681014.1], *Tenacibaculum* sp. from biofilms that induce metamorphosis of marine polychaete [98.82%; MG819702.1]
			*Chryseobacterium* sp. | Candidatus *Amoebinatus massiliae* [100%; AY204874.1], *Chryseobacterium* sp. in goldfish gut [99.76%; MN935216.1]
			*Tenacibaculum* sp. | biofilms that induce metamorphosis of marine polychaete [99.29%; MG819702.1], shrimp gut [99.06%; KF342728.1]
Halanaerobiales [Halanaerobiaceae]	0.55	1	*Halanaerobium* sp. | *Montastraea faveolata* (healthy and diseased coral) [97.64–100%; FJ202891.1, JQ516458.1, FJ203280.1]
Chitinophagales [Chitinophagaceae]	0.48	4	*Sediminibacterium* sp. | tropical urban freshwater [100%; KX968184.1], ureolytic biocementation (carbonate precipitation) in soil [97.64%; MN656428.1]
			Unclassified Chitinophagaceae | *Ciona intestinalis* (ascidian) [97.64%; F799383.1], shrimp gut [96.94%; KP947105.1]
			Unclassified Chitinophagaceae | eutrophic freshwater lake [98.01%; EU273038.1], anaerobic digester [95.75%; MN157568.1]
Entomoplasmatales [Spiroplasmataceae]	0.46	1	Spiroplasma sp. | Arthropod-symbiotic *Spiroplasma* from shrimp [89.07 %; KR349130.1, KY115222.1]
Microtrichales [Microtrichaceae]	0.36	4	Unclassified Microtrichaceae Sva0996 marine group | *Actinobacterium* in *Poecillastra compressa* (deep sea sponge) [95.58%; KF597097.1], Fe-rich hydrothermal sediments [91.15%; FJ905720.1]
			Unclassified Microtrichaceae Sva0996 marine group | *Actinobacterium* in coral reef sediment [99.75%; JN874654.1], *Porites lutea* (coral) [98.27%; KP303904.1]
			IMCC26207 (Microtrichaceae) | Wastewater treatment system [100%; LR634799.1], sediments, lake water [99.75%; MF689304.1, KX367772.1]
Chthoniobacterales [Chthoniobacteraceae]	0.26	1	Candidatus *Udaeobacter* sp. | Mountain forest and soil at CO_2_ spring [100%; MG716938.1, HF952262.1]
Saccharimonadales [unclassified]	0.24	1	Unclassified Saccharimonadales | Soil [96.05%; JQ367084.2], planktonic bacteria [91.36%; Q472788.1]
Bacteria [unclassified]	0.22	1	Unclassified Bacteria| Mollicutes bacterium from ascidians in eel pond [85.55%; EF137401.2], *Anemonia viridis* [86.43%; KC862086.1]
Bdellovibrionales [Bdellovibrionaceae]	0.21	4	*Peredibacter* sp. | biofilm on a copper-based antifouling paint, seawater [99.76%; JN594639.1, MH121376.1], *Favia* sp*.* (healthy and diseased coral) [98.12%; GU472125.1]
			*Peredibacter* sp. | sediments [96.71%; KC925129.1], *Ciona intestinalis* (ascidian) [96.05%; KF799703.1], crab [96.01%; KC917599.1]
			*Peredibacter* sp. | seawater [100%; MH121376.1], *Favia* sp. (healthy and diseased coral) [98.19%; GU472125]
Desulfobacterales [Desulfobacteraceae]	0.19	1	*Desulfatitalea* sp. | *Montastraea faveolata* (coral) [100%; JQ516448.1]
Betaproteobacteriales [Burkholderiaceae]	0.19	4	Burkholderia-Caballeronia-Paraburkholderia | human blood [99.77%; AB374482.1], *Bradyrhizobium* sp. from beech tree roots [99.53%; KX023689.1]
			*Ralstonia* sp. | *Ralstonia pickettii* from soil, pinapple roots, other plants [100%; MT322968.1, LR797737.1, MT341804.1]
			*Bordetella* sp. | *Burkholderia* bacterium from lupins and grass roots [99.77%; JN590346.1, LC031362.1], *Bordetella* sp. from water and sediment of abandoned uranium mine [99.77%; KF441609.1]
***Porites*** **“I” recipient microbiomes**			
**Order [families included]**	**Relative abundance**	**No. of ASVs**	**Lowest taxonomic classification (SILVA) | sources and nearest relatives [% similarity; GenBank accession#]**
Oceanospirillales [Endozoicomonadaceae]	4.64	9	*Endozoicomonas* sp. | *Porites lutea* (healthy coral) [99.77%; KF179706.1, KF179699.1], *Porites compressa*, *Porites lobata* [99.53%; FJ930621.1]
			*Endozoicomonas* sp. | *Porites lutea* (healthy coral) [100%; KF180095.1, KF180125.1]
			*Kistimonas* sp. | *Neofibularia nolitangere* (sponge) [97.44%, EU816849.1], *Alcyonium gracillimum* (soft coral) [96.97%; JF925015.1]
Chlamydiales [Simkaniaceae]	0.10	1	*Simkania negevensis* Z | endolithic bacteria from marine intertidal outcrop [94.4 2%; KT979567.1], sediments from Mariana trench [94.64%; MG580090.1]
SAR324 Marine group B [class:Deltaproteobacteria]	0.05	2	SAR324 clade (Marine group B) HF0200_14D13 | Bacterioplankton [89.05-89.30%; MG875850.1, JN232995.1]
			SAR324 clade (Marine group B) HF0200_14D13 | Bacterioplankton [89.30-89.55%; MG875850.1, JN232995.1]
Nostocales Incertae Sedis	0.01	1	*Oscillatoria* SAG8.92 | endolithic bacteria from marine intertidal outcrop [99.26%; KT973113.1], *Oculina patagonica* and bleached *Muricea elongata* (corals) [99.02%; KU936867.1, DQ917838.1]
Cellvibrionales [Cellvibrionaceae]	0.01	1	Candidatus *Endobugula* sp. | corals in fish farm effluent [96.28%; GQ413096.1], Candidatus *Endobugula glebosa* in *Bugula simplex* (bryozoan) [96.28%; AY532642.1]
Bacteroidales [Marinifilaceae]	0.01	1	*Marinifilum* sp. | *Montastraea faveolata* (coral) [97.64%; FJ202823.1], corals in fish farm effluent [97.41%; GQ413742.1]
Spirochaetales [Spirochaetaceae]	0.003	1	*Spirochaeta* 2 sp. | *Siderastrea stellata* (bleached coral) [95.34%; JF835682.1], *Millepora* sp. (coral) [95.57%; HQ288601.1]

## Discussion

Probiotics could become a supporting intervention to alleviate the coral reef crisis by helping corals to endure increasing temperature stress through administration of beneficial bacteria [4]. In this regard, our experiments combine a field-based and application-focused perspective with the aim to develop probiotics. We tested a coral microbiome transplantation procedure for corals (a.k.a., "CMT", Fig. 1) using two locally and globally important reef-building coral species from the Thai Andaman Sea, *Pocillopora* sp. and *Porites* sp., showing that treatment with a CMT inoculum among conspecifics partially reduced the bleaching response of recipients, as assessed in a short-term heat stress test. We also assessed responses of the recipient microbiomes, which were distinct between the two coral species. In both coral species however, several donor-specific and typically dominant bacterial taxa (i.e., coral-resident bacteria or putative symbionts) were potentially transmitted through the CMT treatment and may be suspected of helping to establish a more heat-resistant recipient coral phenotype.

### Recipients showed reduced bleaching responses under heat stress after inoculation

The first hurdle of performing the CMT was the inoculation procedure which required maintenance of recipients in small volumes of water with limited flow and enriched particle and bacterial loads [37]. This may represent challenging conditions for corals that are most commonly found in clear seawater with regular flow dynamics [38]. Our experiments showed that the procedure did not harm neither of the recipients. Here, bacterial densities of inoculation were all in the range of 10^5^–10^7^ of bacterial cells/mL in bulk and were slightly higher for *Porites* than for *Pocillopora*.

Next and most importantly, heat exposure following the inoculation treatment revealed a higher bleaching resistance in the recipient group compared to the control group that received a sterile-filtered seawater inoculum (i.e., a cell-free control treatment), indicating either a mitigation of stress or a delay of the onset of bleaching. This compares well with observations by Rosado et al. [30] where stress responses were strongly reduced after inoculation with putatively beneficial marine bacterial isolates, but not fully eliminated. Interestingly, the CMT did not benefit photosynthetic resilience, which is in line with the insight that bleaching susceptibility might not be necessarily linked to declines of symbiont performance [39]. Altogether, outcomes indicate that a reduction of stress on the host side of the holobiont was facilitated by the CMT treatment, preventing recipients from bleaching at the same high rates as the cell-free control group.

### Coral-specific bacterial symbiont taxa were transmitted during CMT

Our inoculations had a beneficial effect on recipients' heat resistance, but did not trigger any large-scale restructuring of the microbiome in neither of the two coral species. The question remains whether large microbiome community changes are required for achieving an alternation of the phenotype. Studies have reported both cases, microbiome differences without measurable signs of phenotype changes or the contrary [40–43]. Indeed, substantial reshaping of the microbiome can remain silent when phylogenetically distinct, but functionally redundant bacteria take over niches [44]. On the other hand, we cannot exclude that already small-scale manipulation of the coral microbiome may already lead to phenotypic effects. Our sequencing data captured such small-scale changes that were linked with the reduced coral bleaching response observed, by identifying putative CMT-transmitted bacteria. Most of these donor-specific bacterial species, found in the microbiomes of recipients after inoculation, were members of several typical coral-resident taxa, whose nearest relatives have previously been found in many other coral microbiomes (see references in Table 1). Notably, several of these bacterial species were members of strongly dominant taxa of our coral species’ microbiomes, i.e., a Candidatus *Ampoebophilus* sp. and *Alteromonas* sp. in *Pocillopora* and *Endozoicomonas* sp. in *Porites*; among which Candidatus *Ampoebophilus* is suggested to have co-evolved with the coral holobiont [13] and *Endozoicomonas* is the most prominent candidate coral symbiont known to the coral microbiome research community [45, 46]. These transmitted bacteria originated from donor corals that are naturally coping well with higher environmental stress levels in high variability habitats [36]. Thus, their essential microbiome members may be well adapted to living in a stress-challenged holobiont and are likely to have evolved optimized traits to perform holobiont services more efficiently. Our findings suggest that these bacterial taxa (ASVs) identified by our analyses might more likely integrate into coral microbiomes than other more elusive bacterial species that were also inoculated as part of the CMT inoculum. Based on these results, we underline the importance of future research to elucidate dynamics and interactions of these potential coral symbionts with the host, specifically considering their potentially beneficial effects on coral holobiont health and stress tolerance [4].

Our list of transmitted donor-specific bacteria represents some of the potentially new species that were incorporated into recipient tissues or into the mucus after inoculation. Cells that loosely attach to the corals were excluded through thorough rinsing of the samples with sterile-filtered seawater. However, this list merely represents a list of “candidates,” which the 16S rRNA gene marker, the sequencing effort, and our analysis have been able to capture, while it is important to consider that the use of another marker gene region and a deeper sequencing effort could deliver a more accurate picture [47]. In future microbiome manipulation studies like ours, employment of deep sequencing, the use of full-length 16S rRNA sequences, or metabarcoding at bacterial strain level will very likely prove to be more efficient to capture the processes of bacterial transmission and microbiome community shift [48].

### Coral microbiome dynamics: drivers of stability and flexibility

Microbiome analyses in this study taught us new valuable lessons about coral microbiome dynamics and flexibility (see current perspectives in [49]). Community dynamics in our experiments were most evident and community shifts were significant in the pocilloporid recipients, which responded with an increase of dispersion between individuals, despite the reputation of having a low microbiome flexibility and being coined as a microbiome regulator [14, 41]. In comparison, the community structure remained stable in *Porites* recipients, even though they had experienced a longer lasting and repeated inoculation procedure during CMT. We observed a dynamic *Pocillopora* and stable *Porites* microbiome in our study. The contrary has been so far reported in few other studies where pocilloporid microbiomes were the most inflexible [14, 41], which has been suspected to be a host species-specific characteristic. Interestingly, these inflexible microbiomes were strongly dominated by the bacterial taxon of *Endozoicomonas*. Our study demonstrates the contrast between a flexible pocilloporid microbiome against an inflexible and *Endozoicomonas*-dominated *Porites* microbiome*,* providing the insight that flexibility might be determined by the initial bacterial composition, rather than coral host species. In this regard, our results support the notion that community evenness and bacterial species dominance are drivers for microbiome stability in a holobiont context [50]. Additionally, our sequencing data captured a significant proportion of predatory bacterial taxa, members of *Bdellovibrio* and like organisms “BALOs” [51], in the flexible pocilloporid donor coral and the inoculum. Such micropredators can shape a bacterial community already at low abundances which compares to top-down predator effects in macroscopic ecosystems [52, 53]. In our experiments, species of *Peredibacter* sp. were transmitted to pocilloporid recipients and may have acted as the main drivers of the microbiome changes after inoculation, whereas BALOs were absent in the stable microbiomes of *Porites*. Noteworthy, BALOs constitute a suitable bacterial group to be considered for microbiome manipulation approaches as already implemented in aqua farming projects [54, 55]. Many BALOs are culturable and can protect corals from pathogenic and coral bleaching-associated *Vibrios* [29].

### “Therapeutic agents” in a CMT

Our study comes from a bacterial microbiome-focused perspective hypothesizing that bacterial community changes and specific taxa can be meaningfully linked with the altered phenotype of recipients [56]. This mirrors the perspectives and developments of clinical fecal microbiome transplantation therapies, which has been mostly driven by the human gut microbiome research community and focusing on microbiome community changes that are associated with physiological responses of the recipients [25]. However, to this point, the question for the “therapeutic agents” of microbiome transplantation methods remains not fully resolved [57–60]. A beneficial effect of an inoculation may not solely stem from bacterial input, but other components of the inoculum should be considered.

In corals, a bacterial inoculum serving as an additional food source has been debated [28]. Our CMT inoculum, made from fresh coral tissues, could have indeed offered a food source in form of cell debris, microbial cells, and dissolved organic carbon that could have been ingested by the recipient. It is well known that a significant nutritious input can benefit resilience of corals over a time span of a month [61], but whether a smaller-scale input from an inoculation treatment (as the CMT) could have contributed enough energy reserves to sustain recipient corals through heat stress in our experiment remains unresolved here. Moreover, our current study design does not allow to disentangle the potential therapeutic efficacy of other inoculum components, which aside from bacteria, include biological agents, such as dinoflagellates, other protists, fungi, and phages [9], but also bioactive molecular matter, such as enzymes, signaling molecules, antimicrobials, or regulatory mRNAs [62]. We recommend for future experiments to work with multiple “control” treatments that use modifications and fractions of a CMT inoculum to address the question for the therapeutic agents of such inoculation treatments. Here, in particular, we recommend working with heat-killed inocula and self-inoculation treatments to test for the suspected nutritional benefit and sterile-filtered inocula which can reveal molecular effects stemming from the cell-free supernatant.

### The next steps to further explore the CMT strategy

Our study encompasses experiments performed on a short-term schedule including 1–3 days of inoculation treatments and 1–2 days of acute heat stress exposure. To further develop this method for implementation in coral propagation efforts in a reef restoration setting [2], we emphasize on the importance of a meticulous selection screening for healthy donors to minimize the potential transmission of pathogenic and harmful bacteria or other adverse agents (e.g., environmental pollutants). The important next step will be to test recipients under gradually increasing temperature mimicking a natural bleaching event [36, 63] along with long-term monitoring that includes measuring their recovery potential [64]. Eventually, a reef-reintroduction experiment will be required. The persistence of coral microbiome changes and accompanying physiological effects after CMT or probiotic inoculations needs to be investigated. These effects will need to last for a duration of few weeks, since coral bleaching events typically occur locally due to “short-lived episodes of extreme heat” [1]. On a positive note, bacterial community differences after microbiome manipulation have lasted up to 7 days after treatment for the sea anemone *Nematosella* [65] and a donor footprint including health benefits has been successfully documented in human gut microbiome transplantation recipients for 1–2 years [66].

## Conclusions

We draw the final conclusion from a microbiome-focused perspective. Approaches that investigate the probiotic potential of microbiome manipulation, such as several previous coral probiotic studies [30, 31, 67] and the CMT concept developed in our study (Fig. 1), are founded on the hypothesis that exposure of the holobiont to beneficial bacterial consortia can be applied to increase health and resilience. This could be a result of a shift towards a more efficient microbiome community composition through the proliferation of beneficial bacteria that were already present within the microbiome or the incorporation of “entirely new” taxa or strains that provide additional functions helping the holobiont cope with stress. Our 16S rRNA gene metabarcoding data suggests a possible scenario, which could have been the uptake of a specific “new” variant of a typical coral-resident or “symbiotic” bacterium that can provide holobiont services in a more efficient manner under stress conditions than the native “symbionts.” These results further suggest that such bacterial taxa should spcifically be included into the scope of coral probiotics developments. Our CMT experiments demonstrated that coral bleaching resistance can be positively influenced in a field-based setting. Hence, this strategy might reveal itself as a feasible approach to support coral heat resistance and find application to enhance efficiency of certain coral propagation and restoration efforts. If scaling up the method will not turn out to be feasible any soon, another perspective is that CMT can serve as an elegant manipulative tool which could help further advance the identification of probiotic bacterial species and strains, which then could be further developed into a probiotic inoculation treatment *sensu* [4, 30].

## Materials and methods

### Coral collection sites, taxa, and maintenance

Collection sites were located in the Andaman Sea, Thailand (Fig. 2a, see details in *Supplementary Material and Methods*). They were purposely chosen based on their local environmental differences and implications for coral heat tolerance [36, 68]. We refer to the collections sites as “LowVar” for sites of low temperature variability and “HighVar” for sites of high temperature variability (Fig. 2b). Fragments from visually healthy colonies of *Pocillopora* sp. (April 2018) and *Porites* sp. (November 2018) were collected. Both corals represent two distinct coral ecotypes (branching vs. massive morphology) and are abundant and ecologically significant coral species in Thailand [69]. All experiments were performed inside four 40 L tanks (Tables S6-7).

### Heat tolerance assessment

We assessed coral heat tolerance by employing short-term heat tolerance assays to quantify stress responses in a high-throughput manner. We tested a suite of colonies from the different sites. Ten colonies from the “LowVar” and 9 from the “HighVar” site were screened in the genus *Pocillopora*, and 14 colonies from the “LowVar” and 12 from the “HighVar” site in the genus of *Porites*. Two fragments from each colony were randomly distributed among the two treatments, “34 °C” and “29 °C” (tanks *N* = 2). The “34 °C” treatment was established by ramping temperatures from 29 to 34 °C for 4 h, holding at 34 °C for 5 h (*Pocillopora*) or 6 h (*Porites*), and decreasing temperatures to ambient 29 °C within 4 h (Fig. S1 A-B). Afterwards, all corals were maintained at ambient temperature for 10–11 h until the next day. While *Pocillopora* corals were subjected to one 34 °C-heat peak, resulting in a full experiment of 24 h (see experiment schedule in Fig. S1 A), *Porites* corals were exposed twice under 34 °C-heat peak resulting in a heat tolerance assay of 72 h in total (Fig. S1 B).

### Coral microbiome transplantation experiments

Based on the outcomes of the heat tolerance assessment, colonies from the “LowVar” site were designated to be recipients. For the coral microbiome transplantation (CMT) experiments, we collected four fragments per colony of *Pocillopora* (colony *N* = 5) and *Porites* (colony *N* = 10). Each experiment consisted of two parts: (i) the inoculation phase with two treatment groups, “I” (i.e., recipients of a CMT inoculation) and “C” (i.e., control group receiving a FSW inoculation), and (ii) the reassessment of heat tolerance resulting in four experimental groups, i.e., “I x 29 °C,” “I x 34 °C,” “C x 29 °C,” and “C x 34 °C.” We designated colonies from the “HighVar” sites to be donors and collected six *Pocillopora* fragments from the west shore of Racha Island (colony *N* = 3, two fragments per colony) and 18 *Porites* fragments from Panwa reef flat (colony *N* = 6, three fragments per colony). Inocula were prepared 2 h before each inoculation event by homogenizing the tissues of each donor fragment according to established protocols for coral pathogen transmission ([37], *Supplementary Material and Methods* and Fig. S10)*.* Four fragments of each recipient colony were randomly distributed among the four experimental tanks to commence the inoculation phase. Inoculations were performed at 29 °C. Current pumps and aeration were interrupted and the seawater volume was reduced to 8 L (i.e., 6 cm water level). Next, PVC tubes (height 7 cm, ø 8 cm, volume 350 mL) were placed around each coral fragment to create a semi-enclosed microenvironment to which inoculation shots were added [70]. The control treatments consisted of shots of filtered seawater (FSW 0.2 μm) that were prepared without the addition of donor-tissue material. Pocilloporid recipients each received one inoculation shot over 24 h, while *Porites* recipients each received three shots repeated every 24 h over 3 days (Fig. S1 C-D). Corals were incubated with the inocula inside the tubes for 30 min (*Pocillopora*) or 2 h (*Porites*). Subsequently, PVC tubes were removed, water flow and aeration were switched back on, and tanks were filled up to 40 L, further diluting bacterial densities. After 24 h, a regular seawater exchange (50% twice a day) was continued. Differences between the two inoculation procedures stem from logistic limitations such as availability of coral material. Following the inoculation phase, heat stress tolerance was reassessed in both recipient groups. Heat tolerance assays were performed as described above with a minor modification of exposing *Pocillopora* recipients at 34 °C for 7 instead of 5 h.

### Coral response variables

Stress response variables were measured for each fragment before and after each experimental part. First, a bleaching score was determined as a measure of dinoflagellate symbiont cell density in coral tissues. The same observer visually categorized individual fragments on the scale from “1” (bleached) to “6” (healthy), recording a minimum and maximum score (“Coral Health Chart,” Coral Watch, reefquest.org, [71]). Second, photosynthetic efficiency of symbionts was assessed in light-adapted fragments measuring effective quantum yield (Φ PSII = (Fm’ – F)/Fm’ = ΔF/Fm’, [72]) using a pulse amplitude-modulated fluorometer (Diving-PAM, Walz, Germany).

### Microbiome sequencing

Coral and seawater samples were collected at three time points during the CMT experiments, “start” of inoculation, “end” of inoculation, and “end” of heat tolerance reassessment (Fig. S1 C-D). DNA was extracted following established protocols. The variable region V3–V4 of the 16S rRNA gene (357F [5′CCTACGGGAGGCAGCAG′3], 806R [5′GACTACHVGGGTWTCTAAT′3]) was amplified and sequenced [73], including quality control (QC) samples, i.e., PCR and DNA extraction kit blanks. Amplicon library preparation and sequencing were performed at the IKMB Sequencing Center (University of Kiel, Germany). Data were delivered by two Illumina runs and processed separately using QIIME2 v2019.7 integrating DADA2 [74]. Amplicon sequence variants (ASVs) from both runs were merged prior to classification with SILVA v132 [75]. Sequence reads from QC samples were used to identify contaminants and clean up the data (Dataset S4). Sequencing data are available in the NCBI Sequence Read Archive (SRA) under accession number PRJNA647757. A rarefied data set was generated after determination of a suitable subsampling depth using rarefaction tools as implemented in QIIME2. Rarefaction curves were plotted using function *rarecurve* (R package *vegan* v2.5-6). A filtered data set, “filt-10,” was created from the full data excluding rare ASVs with a total read abundance of <10. Details are provided in the *Supplementary Materials and Methods* and Dataset S5.

### Statistical analysis

∆-values of coral response variables (end–start of each experimental part) were used for analyses. Effects of (i) temperature treatments (“34 °C” vs. “29 °C”) within the site of origin (“HighVar,” “LowVar”), (ii) inoculation (“I” vs. “C”), and (iii) subsequent temperature treatment of inoculated recipient corals (“34 °C” vs. “29 °C”) were evaluated using *dabestR* v0.2.3 6 [76] and linear mixed effect models (*nlme* v4 3.1-148 and *lme4* v1.1-23 package). Where applicable, coral colony genotype was used as a random factor.

Microbiome α- and β-diversity analyses were performed on the rarefied data using *phyloseq* v1.32.0 and *vegan* v2.5-6 in R. We compared (i) coral and seawater microbiomes pooled across time points (“*Pocillopora*,” “*Porites*,” “seawater tank,” “seawater source”), (ii) coral groups at experiment “start” (“donor,” “inoculum,” “recipient start group”), (iii) at the “end” of inoculation, and (iv) at the “end” of heat tolerance reassessment. α-diversity metrics were analyzed by *dabestR* v0.2.3, ANOVA or Kruskal-Wallis, and generalized linear or linear mixed effect models where suitable. Dissimilarities (Bray-Curtis) and homogeneity of variances were tested by PERMANOVA with 9999 permutations using *adonis2* and by PERMDISP using *betadisper* function, respectively. Pairwise tests followed respectively, *pairwise.perm.manova* or Tukey HSD test through *betadisper*. The “filt-10” data set was used to create stacked bar plots at the bacterial species level (showing the most dominant species: relative abundance >10%). *UpSetR* (v1.4.0 [77];) analyses were performed with “filt-10” data to (i) characterize shared bacterial taxa in the coral and seawater microbiome, (ii) identify exclusively unique taxa in the inoculum, and (iii) capture bacteria uniquely shared by the inoculum and the recipients after inoculation.

## Supplementary Information

**Additional file 1. Supplementary materials.**

**Additional file 2: Dataset S1. Analysis table of coral response variables. ‘**LowVar’ site = Racha Island east shore, ‘HighVar’ site (*Pocillopora*) = Racha Island west shore, ‘HighVar’ site (*Porites*) = Panwa reef flat; heat tolerance (HT) assessment treatments: ‘34 °C’ and ‘29 °C’; Inoculation treatments: ‘I’ = inoculation; ‘C’ = sterile-filtered seawater (FSW) control group; n = replicate fragment numbers; mean difference = mean difference between start and end of a treatment; SD = standard deviation; SE = standard error; CI = confidence interval.

**Additional file 3: Dataset S2. Analysis tables of microbiome data.** Count tables are provided for (**A**) ‘filt-10’ data and (**B**) rarefied data (subsampled to 4 000 reads). Tables enclose read abundance counts per amplicon sequence variant (ASV), experiment metadata (i.e., treatment groups), and SILVA classification. TYPE = sample type; POC = *Pocillopora*; POR = *Porites*; SW = seawater. Timepoints include: t1 = start, t2 = end of inoculation, t3 = timepoint of water collection from the source tank during heat tolerance (HT) reassessment, t4 = end of HT reassessment. I = inoculation; C = FSW control group; H = heat exposure treatment 34 °C; A = ambient temperature treatment 29 °C.

**Additional file 4: Dataset S3. Full table of potentially transmitted bacteria in the (A)** ***Pocillopora*** **and (B)** ***Porites*** **experiment.** Amplicon sequence variants (ASVs) exclusively shared between the inoculum and the ‘I’ recipient’ group after inoculation are provided each with their respective SILVA based taxonomy and sequence. Read abundances for each potentially transmitted ASV show its occurrence within the different treatment groups. ASVs are marked in ‘green’, when also detected in the donor samples. Those, also detected in seawater samples, are marked in ‘blue’. ASVs are marked in red, when detected in the ‘I’ recipient group at the end of heat tolerance reassessment (i.e., ASVs that persisted within the recipients’ microbiomes until the very end of the experiment).

**Additional file 5: Dataset S4. Quality control samples and clean-up of microbiome data.** Tables show host-origin and contaminant amplicon sequence variant (ASV) sequences that were removed from the microbiome data set prior to downstream analyses. (**A**) Table shows ASV sequences of host-origin as matched with GenBank (NCBI). (**B**) Table shows ASVs identified as contaminants using DNA Extraction Kit and PCR blank samples. Stacked bar charts show bacterial community compositions of (**C**) extraction kit blank samples and (**D**) PCR blank samples. Additionally, scoring tables for contaminant ASVs are shown.

**Additional file 6: Dataset S5.** Protocol of raw read processing using QIIME2 V2019.7.

## Data Availability

Sequence data generated in this study are available under NCBI BioProject ID PRJNA647757. Source data underlying analyses are provided with the Supplementary Dataset files, i.e., analysis tables for phenotypic response variables are provided in Dataset S1; ASV count tables including metadata, corresponding taxonomic classification, and read sequences are presented in Dataset S2; and quality control samples and decontamination process of the data are presented in Dataset S4. The raw read processing protocol using QIIME v2019.7 including summary statistics is provided in Dataset S5.

## Abbreviations

CMT: Coral microbiome manipulation
FSW: Sterile-filtered seawater
ASV: Amplicon sequence variant
QC: Quality control
nMDS: Non-metric Multidimensional Scaling
PERMANOVA: PERMutational multivariate ANalysis Of VAriance
BETADISPER: BETA-diversity analysis of multivariate homogeneity of group DISPERsions (variances)
