# Supplementary material for: Towards enhancing coral heat tolerance: a “microbiome transplantation” treatment using inoculations of homogenized coral tissues
Source: Microbiome. 2021 May 6;9:102. doi: 10.1186/s40168-021-01053-6 (PMC8103578; doi:10.1186/s40168-021-01053-6)
Supplement: Supplementary file 2 — Additional file 1. Supplementary materials. [file 40168_2021_1053_MOESM2_ESM.pdf]

Supplementary Materials

Towards enhancing coral heat tolerance: a “microbiome transplantation” treatment using inoculations of homogenized coral tissues

Short Title: Coral microbiome transplantation

Authors

Talisa Doering[1], Marlene Wall[1], Lalita Putchim[2], Tipwimon Rattanawongwan[2], Roman Schroeder[1], Ute Hentschel[1], Anna Roik[1*]

Affiliations

[1]GEOMAR, Helmholtz Centre for Ocean Research, Kiel, Germany

[2]Phuket Marine Biological Center (PMBC), Phuket, Thailand

[*]Corresponding author:

Email: aroik@geomar.de

The PDF file includes:

- Supplementary Materials and Methods
- Figs. S1 to S11
- Tables S1 to S7
- Captions for Excel Files: Datasets S1 to S4
- Caption for Word Document: Dataset S5

Supplementary Materials and Methods

Coral collection sites and taxa

Collection sites were chosen based on known differences of environmental variability. Two sites were exposed to remarkable environmental fluctuations ('HighVar' sites: Racha Island west shore at 15 m depth [7.595530 N, 98.354320 E] and Cape Panwa reef flat at 0-2 m depth [7.802553 N, 98.405855 E]; Fig. 1). One site was sheltered and provided near-optimal, stable, shallow reef conditions ('LowVar' site: Racha Island east shore at 15 m depth [7.598910 N, 98.373100 E]). In situ temperature was continuously monitored in each site two weeks prior to each experiment (HOBO Pendant Data Logger, Onset, USA, Fig. 2 C-D).

On the western shore of Racha Island, internal wave forcing causes strong variability through frequent upwelling of deep, cool, and nutrient rich water onto the shelf ('HighVar', 15 m depth, [1–3]), whereas the shallow, intertidal reef flat in Panwa features strong diel fluctuation of temperature and light regimes as well as temperature extremes with weekly averages above 31 °C (0 - 2 m depth, [4]). The eastern shore of Racha Island is sheltered from the impact of internal waves and ocean forcing, thus offers mostly stable environmental conditions, representing a low variability site ('LowVar', 15 m depth).

Coral maintenance

The coral maintenance facility was located at Phuket Marine Biological Center (Cape Panwa, Phuket, Thailand) within two hours by speed boat from the most distant sampling site. Collected coral fragments (*Pocillopora*: length ~ 5 cm; *Porites*: ø ~ 6 cm) were maintained in two large 500 L flow-through tanks with a flow rate of 2.8 ± 1.31 L/min and the average ambient *in situ* temperature of the season for 2-12 days before used in the experiments. A 500 L source tank constantly supplied 5 µm-filtered seawater from the reef adjacent to the facilities and temperature was held at constant 29.43 ± 0.32 °C using a chiller, a heater, and a temperature-control device (Aqua Medic Titan 1500 Chiller, Germany; Titanium Heater 100 W, Schego, Germany; Temperature Switch TS 125, HTRONIC, Germany). During experiments, i.e., heat tolerance assessment and coral microbiome transplantation (CMT), the large flow-through tanks served as temperature-stabilizing baths for four 40 L experimental tanks, two per water bath. Experimental tanks were supplied through daily manual water change (twice daily 50%) from the source tank. Each experimental tank was equipped with a temperature-control device, one heater, air supply, a small current pump, and a temperature logger (Titanium Heater 100 W, Schego, Germany; Temperature Switch TS 125, HTRONIC, Germany; HOBO Pendant Temperature/Light 8K Data Logger, Onset, USA; Koralia nano 900 L/h, Hydor, Italy). Each of the four experimental tanks as well as the flow-through tanks were equipped with LED lights (135 W, Hydra Fiftytwo HD LED, Aqua Illumination, USA) that mimicked the average light conditions of the sampling sites. In particular, due to different light requirements for *Porites* from the Racha island reef site at 15 m and from the shallow reef flat at 0-2 m, heat tolerance assays were performed separately under adjusted light levels for these corals. Tanks were monitored regularly by measuring a suite of environmental parameters (temperature, oxygen, light intensity, and salinity). Briefly, temperature was measured continuously with loggers (HOBO Pendant® Temperature/Light 64K Data Logger, Onset, USA). Other parameters were monitored at regular time intervals, i.e., photosynthetically active radiation (PAR) measured by a quantum meter (MQ-210 Underwater Quantum Meter, Apogee Instruments, USA), dissolved oxygen and salinity measured by a hand-held multimeter (Multi3430, FDO®925, and TetraCon®925, WTW, Germany). Physico-chemical parameters are provided in Tables S6-7.

Inoculum preparation and bacterial cell counts

Whole fragments (*Pocillopora*) or scrapes of tissue of whole fragments (*Porites*) were vortexed each in a tube with sterile glass beads (ø 2.7 mm) and 15 or 35 mL filtered seawater (FSW 0.2 µm) for 1 or 3 min, respectively (Fig. S10 A). On each inoculation day, tissue homogenates were pooled and divided into "inoculation shots" of 8 mL (*Pocillopora*) and 10 mL (Porites). The same procedure, without adding donor material, was carried out for the FSW-inoculum for the control treatment. Each inoculum pool was sub-sampled to extract DNA for microbiome analysis (-80 °C storage) and for bacterial cell counts.

Frozen inoculum samples (-80 °C, 20% glycerol) were thawed at 4 °C, vortexed for 10 s at maximum speed, fixated using formaldehyde (FC 3.7%), and stored at 4 °C until further use. Sample dilutions, 10^{-2} and 10^{-3}, were prepared using 0.22 µm-filtered seawater (FSW) and digested with Trypsin/EDTA (FC 0.2%, Gibco, NY) at RT for 1 h [5]. Trypsinated dilutions were stained with 4,6-Diamidino-2-phenylindole (DAPI, FC 0.7 µg ml^{-1}) and incubated for 30 minutes at RT in the dark. Next, dilutions were pre-filtered (<133 mbar) through a 0.8 µm polycarbonate membrane (Osmonics, CA, USA) using 5 mL additional FSW and washed with another 10 mL FSW. Flow-through was filtered (<133 mbar) onto a black 0.2 µm membrane filter (Merck Millipore Ltd., IRL) and washed with 10 mL FSW. Both filters were washed (3 mL 70% EtOH), mounted onto a glass slides (Citifluor Ltd., UK), and stored at 4 °C. The area of the counting grid within the ocular (DMi8 Microscope, Leica) was measured and nine segments were determined to be counted for each filter. Duplicate counts per filter were averaged. The final counts from both filters were added up per sample dilution and cell densities calculated as cells mL^{-1}. Averages and standard deviations were calculated from the two sample dilutions.

Microbiome sampling, DNA extraction, and amplicon sequencing

Throughout the two experiments microbiome samples were carefully collected from the fragments employed in the experiments using sterile clippers (*Pocillopora*: 1-2 cm clip of each fragment) or sterile peelers (*Porites*: scrape ø 1-2 cm). Prior to collecting the tissue, fragments were rinsed thoroughly with FSW (0.2 um). Then samples were flash frozen in liquid nitrogen. *Porites* 'start' samples were taken from extra coral fragments which were collected in the reef sites from the same colonies during coral collection. Tissue sampling was always performed after the measurements of coral response variables. At the sampling timepoints, seawater samples (1 L) were collected from each experimental tank using sterile cubitainers rinsed with 10% bleach/chlorine solution and MilliQ water. Seawater samples were vacuum-filtered over a 0.2 μm filter (Durapore PVDF filter membranes, Merck, Germany), shock frozen and stored in liquid nitrogen, and subsequently stored at -80 °C. DNA extractions followed a modified Qiagen Allprep DNA/RNA column extraction kit protocol. Filters were preprocessed by thawing (RT for 5 min) and refreezing (-20 °C for 5 min), repeating the cycle 3 times to promote cell lysis, then sliced into stripes, using a sterile scalpel before homogenization [6]. Next, for coral and filter samples, further modifications included the use of lysis tubes (2 mL Lysing Matrix E, MP Biomedicals, USA), bead-mill homogenization (2 x 1 min 30 Hz, Qiagen TissueLyser II, Germany), and centrifugation for 3 minutes at 15 000 rcf, before the clear supernatant was processed following the manufacturer's instructions, adding a second washing step prior to elution of DNA from the column. For library preparation 10-15 ng of DNA were used and the amplification performed with a Phusion HS II High-Fidelity DNA polymerase (0.5 Us) in a dual-barcoding approach [7]. Primer pair 357F [5´CCTACGGGAGGCAGCAG´3] and 806R [5´GACTACHVGGGTWTCTAAT´3] was employed at 0.28 μM. PCR cycling conditions were as follows: 30 s at 98 °C; 30 × [9 s at 98 °C, 60 s at 55 °C, 90 s at 72 °C]; 72 °C, 10 min; 10 °C on hold. PCR-products were normalized using the SequalPrep Normalization Plate Kit (Thermo Fischer Scientific, Waltham, MA, USA), pooled in equimolar amounts, and sequenced on the Illumina MiSeq v3 2x300bp with 20% PhiX (Illumina Inc., San Diego, CA, USA). Quality control samples (QC) were included, i.e., negative (DNA extraction blanks and PCR blanks) and positive controls (#ZRC 190811, ZymoBIOMICS Microbial Community DNA, Zymo Reseach).

Amplicon raw data processing

Amplicon data produced in two Illumina runs were demultiplexed based on 0 mismatches in the barcode sequences. Raw sequence data was processed using a *QIIME2* V2019.7 pipeline. First, PCR primer sequences were removed (*cutadapt,* [8]) and quality of paired-end sequence reads assessed and truncation parameters set at a read quality of Qscore ≥ 20 (*demux*). Assembly of reads, denoising, and generation of bacterial amplicon sequence variants (ASVs) were carried out using the *DADA2* plug-in [9] under default settings, truncating poor quality bases of the forward read at 277 bp and reverse read at 220 bp resulting in an contig overlap of 31 bp for the first library, and truncating the forward read at 278 bp and the reverse read at 230 bp (i.e., overlap of 42 bp) for the second library. This step removed 31% and 26% of sequences from the two libraries, respectively. Now, libraries were merged ('*feature-table'* merge options). A naïve-Bayes classifier object was trained based on the 16S region V3-V4

and SILVA database V132 (99%; [10]) and subsequently employed for the classification of the sequences (*classify-sklearn, feature-classifier;* [11, 12]). Unassigned, mitochondrial, archaeal and chloroplast reads were removed (*feature-table*, *filter-features*). ASV count tables were exported as 'biome' files to be also used in R (*export*, and *biom convert*).
Contaminant bacterial taxa aka amplicon sequence variants (ASVs) were identified through examination of four PCR negative control samples (10,080 reads over 31 ASVs with 95 – 8 854 reads per sample) and 13 DNA extraction kit blank samples (14,328 reads over 92 ASVs and 32 – 5,841 reads per sample). ASVs were scored as contaminants, once they occurred in >1 sample and had a relative abundance higher than 5% or 1% within all control samples, respectively (i.e., a read count of 200-500). 10 contaminant ASVs resulted from PCR negative controls and 27 from kit blank controls (in total 33 contaminant ASVs, Dataset S3). Next, to further identify and exclude coral origin sequences, 241 ASV sequences showing no higher classification level than 'domain: Bacteria' (SILVA database) were compared with GenBank (https://www.ncbi.nlm.nih.gov). 96 such sequences were identified as coral origin sequences. The lists contaminant ASVs and coral origin sequences were subtracted from the full data set in a final clean up step in R environment.

Amplicon data overview
After denoising, classification, and removal of unclassified reads (*QIIME2*) the full 16S rRNA gene amplicon data had 2,560,337 reads across 9,593 amplicon sequence variants (ASVs) and 312 samples with an average of 8 206 reads per sample (including coral and seawater samples from two CMT experiments and DNA-extraction kit blanks). After removal of further unrelated sequences (bacterial contaminants and sequences of coral host origin) samples reached an asymptote at subsampling depth of 4,000 reads, while retaining essential replicate samples. Seawater samples collected from the source tank did not reach asymptote at 4,000 reads and were excluded from α- and β-diversity analyses (three samples from the *Pocillopora* experiment and two samples from the *Porites* experiment, Fig. S3). The data set contained 840,000 reads over 7,177 ASVs and 210 samples after rarefying. In parallel, the non-rarefied data set was filtered by removal of rare ASVs (< 10 reads,'filt-10') resulting in 2 335,885 reads over 4,604 ASVs and 293 samples (for ASV count tables see Dataset S1 This filtering translates to the removal of 0.01% of total reads and 51% of all ASVs and demonstrates a significant proportion of rare ASVs in the sequencing data.

Supplementary Figures

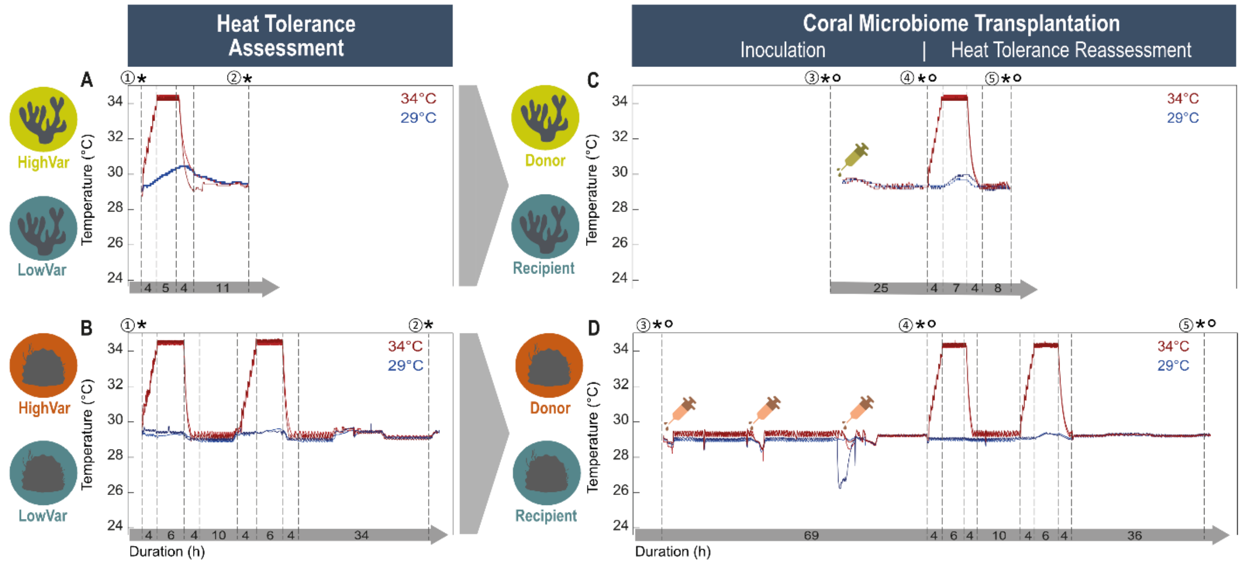

Fig. S1 Temperature profiles of experimental treatments and sampling timepoints. (A-B) Temperature profiles of heat tolerance assays were conducted to identify suitable donors and recipients. Temperature treatments were adjusted accounting for the specific environmental sensitivity of each coral genus: *Pocillopora* was exposed to a single heat-peak over one day; *Porites* required two heat-peaks over two days to show a heat stress response. **(C-D)** Temperature profiles during coral microbiome tranplantation experiments: The inoculation phase was performed at 29 °C and subsequent heat tolerance reassessment peaked at 34 °C. Inoculation was performed once for (**C**) *Pocillopora*, and repeated over three days for (**D**) *Porites*. Timepoints of coral response *measurements* and sample collection are indicated: Start (①) and end (②) of heat tolerance assessment; start (③) and end (④) of inoculation, end (⑤) of heat tolerance reassessment. Types of data and sample collection: ✱ = photosynthetic efficiency and bleaching score measurements; ○ = DNA sampling; syringe icon = CMT inoculation event; branching coral = *Pocillopora* sp.; massive coral = *Porites* sp.; light green = ‘HighVar’ west shore corals; orange = ‘HighVar’ reef flat corals; teal = ‘LowVar’ east shore corals; blue line = ambient ‘29 °C’ treatment; red line = heat stress ‘34 °C’ treatment.

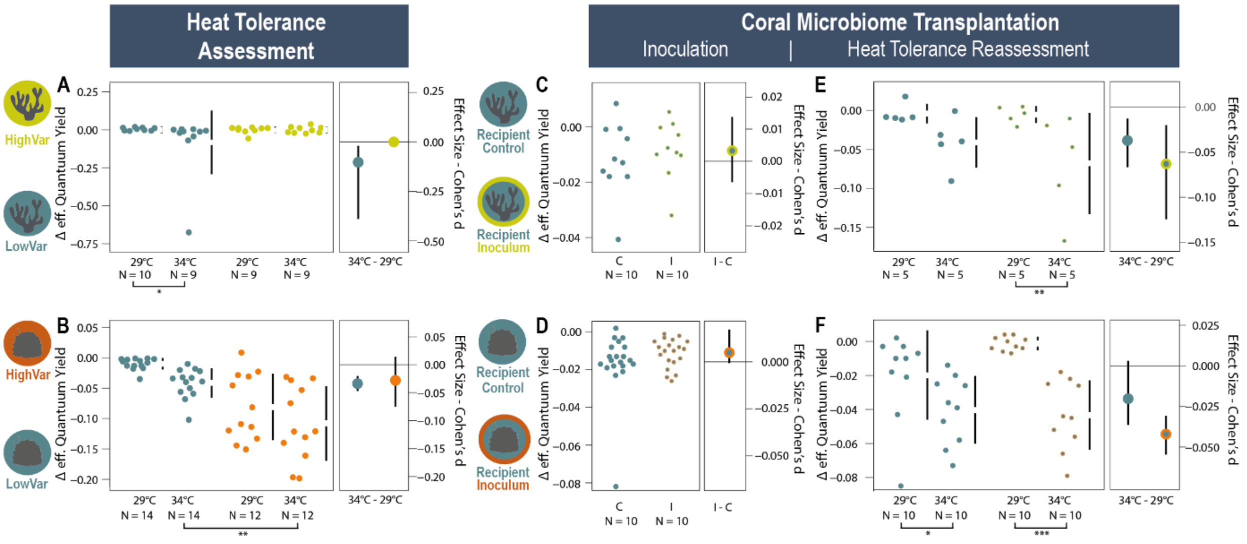

Fig. S2 Responses of effective quantum yield during heat tolerance assessments before and after the coral microbiome transplantation experiments. (**A-B**) Effects of temperature ('29 °C' vs. '34 °C') on the photosynthetic efficiency of corals from sites of low and high environmental variability ('LowVar' and 'HighVar') are compared. (**C-D**) Next, data shows effects of the handling procedure during inoculation ('I' = inoculation group vs. 'C' = sterile-filtered seawater (FSW) control group). Subsequently, (**E-F**) the temperature effect on the photosynthetic efficiency of the recipient group and the FSW control group are shown. Plots visualize Δ- effective quantum yield (i.e., the difference in photosynthetic efficiency at end – start of each experimental part). Swarm plots (left side plot) show raw data points and Cumming estimation plots (right) depict the effect sizes as the mean differences between the treatment groups using Cohen's d and a 95% confidence interval. Significant differences are indicated by connecting lines ($p < 0.001$***, < 0.01**, < 0.05* from generalized linear/linear mixed effect models). Vertical error bars = 95% CI; N = individuals per treatment group; Branching coral = *Pocillopora* sp.; massive coral = *Porites* ***sp.***; light green = 'HighVar' west shore corals; orange = 'HighVar' reef flat corals; teal = 'LowVar' east shore corals; colored circles represent the donor inoculum used: light green = 'HighVar' *Pocillopora* donor, orange = 'HighVar' *Porites* donor.

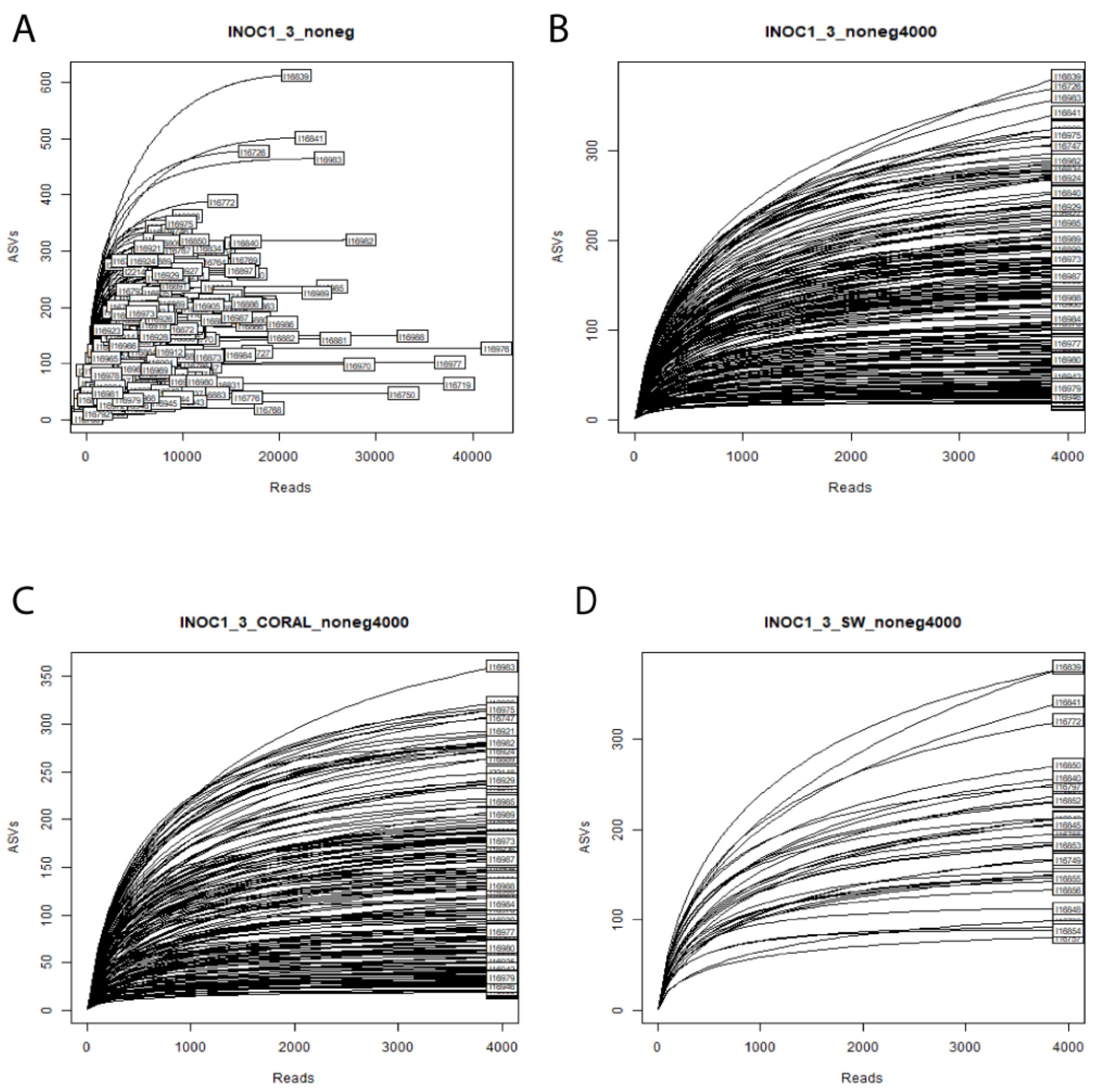

Fig. S2 Rarefaction curves for 16S rRNA gene amplicon data. (**A**) Rarefaction shows all samples (coral and seawater) of the data set after decontamination and clean up (quality control samples excluded). (**B**) Data rarefied to 4 000 reads are shown and further separated by sample type, (**C**) coral and (**D**) seawater. X-axis = read count; Y-axis = observed ASVs (amplicon sequence variants).

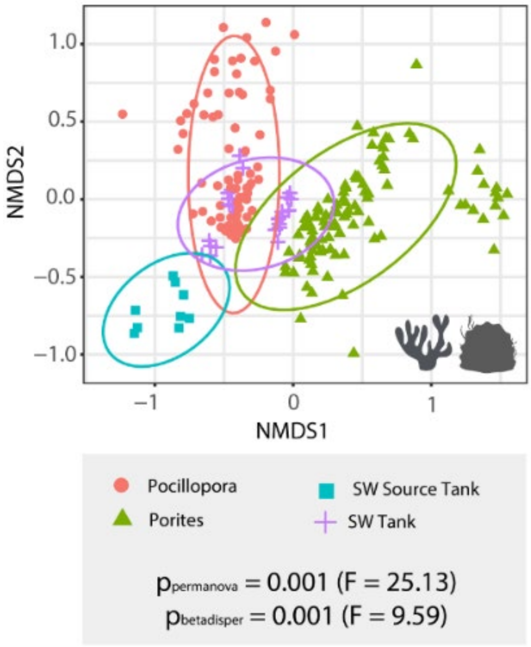

Fig. S3 β-diversity of bacterial communities across both experiments. Differences between the microbiomes of the two coral species (*Pocillopora* sp. and *Porites* sp.) and the respective seawater (SW) sources ('SW experimental tanks' and 'SW source tank') are shown using non-metric Multidimensional Scaling (nMDS) and Bray-Curtis dissimilarities. Group differences based on dissimilarities and dispersion were tested using PERMANOVA and BETADISPER analysis and p and F values are reported (R package *vegan*).

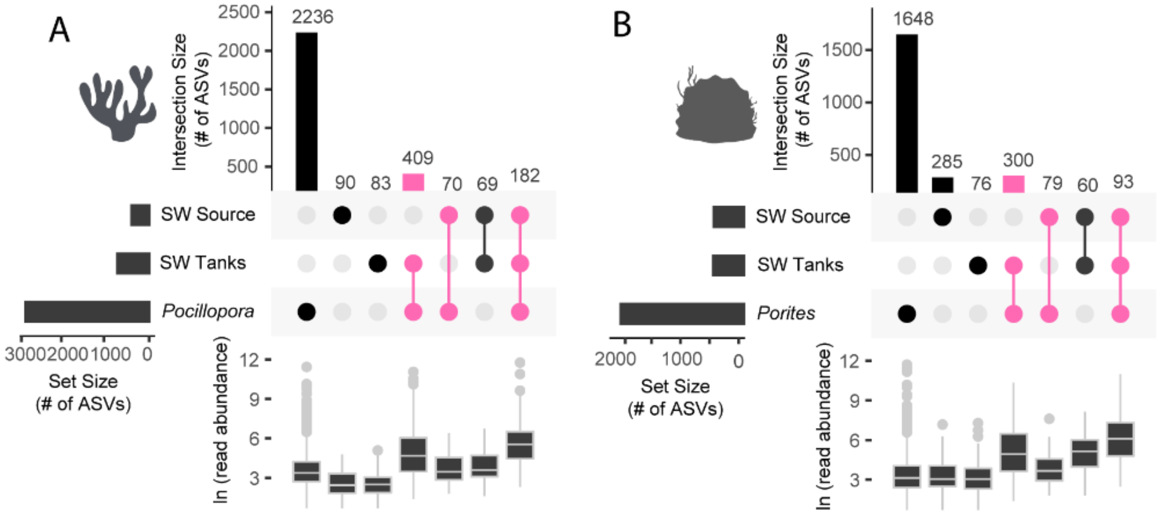

Fig. S5 Shared and unique amplicon sequence variants (ASVs) of coral and seawater. Analyses were performed using *UpsetR* to visualize unique and shared sets of ASVs between the coral microbiomes and the two seawater (SW) sources, i.e., 'SW experimental tanks' and 'SW source tank'. Set sizes are indicated by horizontal bars, unique and intersection group sizes are indicated by vertical bars. Intersections including coral sample sets are highlighted in pink. A boxplot depicts the ln-transformed read count per set, as an indicator for their abundance (**A**) *Pocillopora* and (**B**) *Porites* experiment.

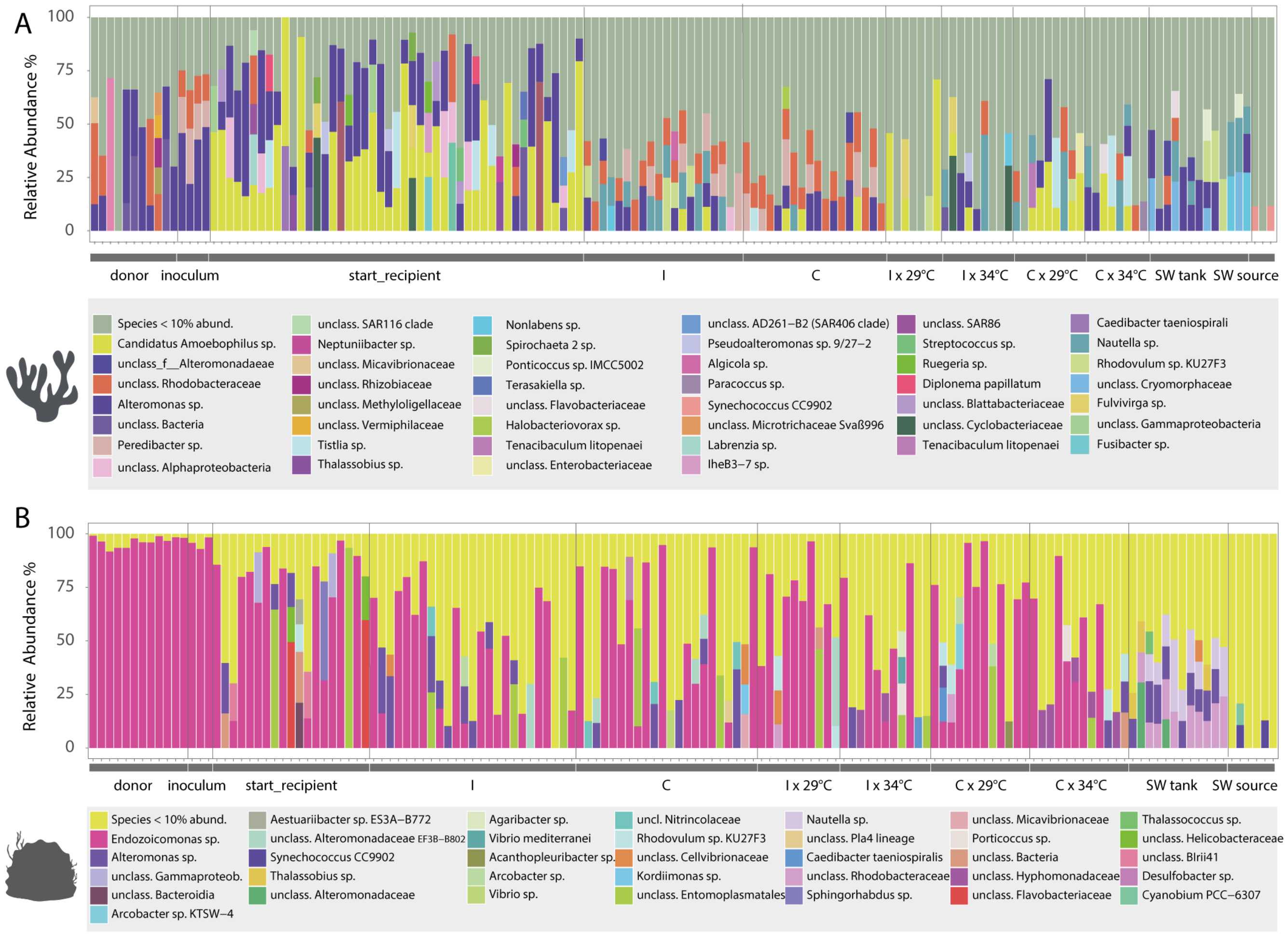

Fig. S6 Bacterial community compositions highlighting the most dominant bacterial species of all coral and seawater samples of the coral microbiome transplantation experiments. Stacked bar plots summarize the community compositions at bacterial species level (SILVA database, bootstrap > 80) depicting the dominant species. Lower abundant species are grouped in one category (< 10% relative abundance). Relative abundances are shown per bacterial species and sample. Sample groups are labeled on the x-axis. (**A**) *Pocillopora* sp. and (**B**) *Porites* sp. experiment. 'I' = recipients of inoculation; 'C' = sterile-filtered seawater (FSW) control group; '29 °C' = ambient temperature; '34 °C' = heat stress treatment.

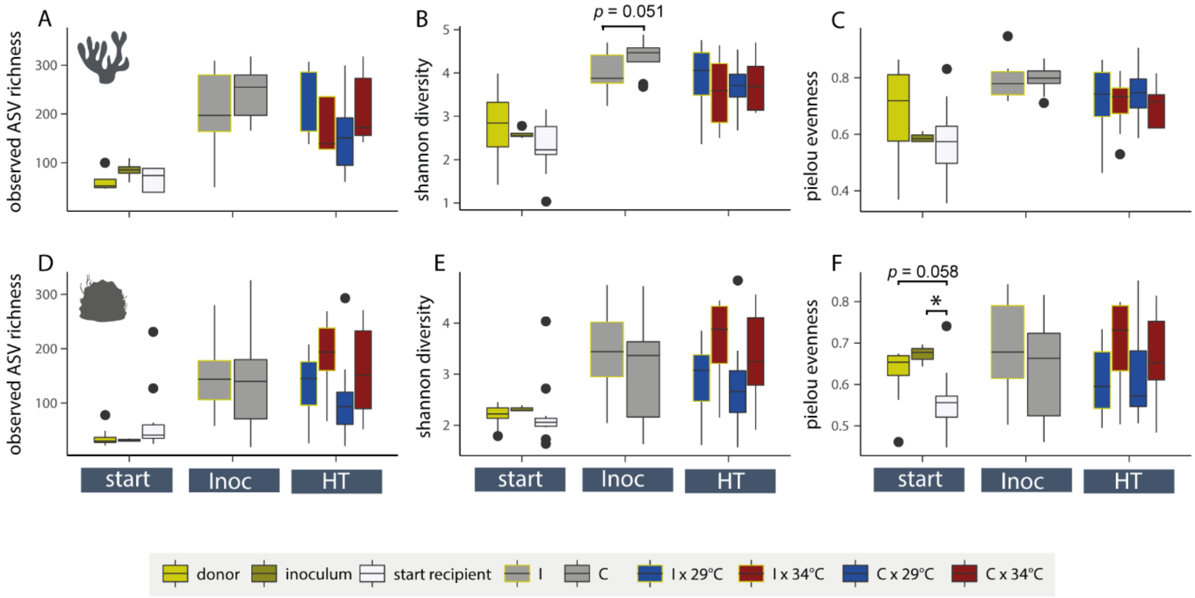

Fig. S7 α-diversity of coral microbiome communities throughout the coral microbiome transplantation experiment. Boxplots visualize three α-diversity metrics: Observed ASV richness, Shannon diversity index, and Pielou's evenness. Data are grouped along the x-axis according to the three experiment parts 'start'= experiment start, 'Inoc' = end of inoculation procedure, and 'HT' = end of heat tolerance reassessment. (**A-C**) *Pocillopora* sp. and (**D-F**) *Porites* sp. experiment. Significant differences: $p < 0.05$*.

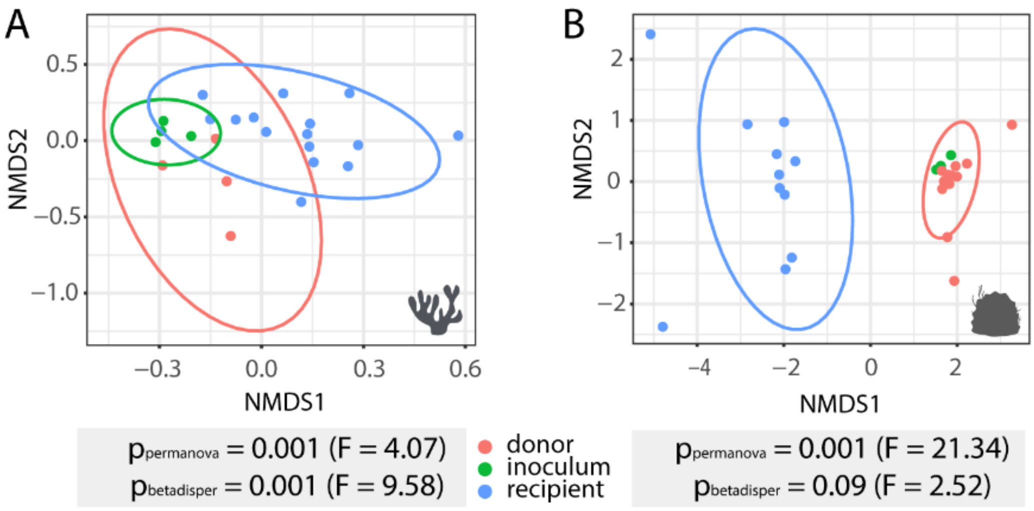

Fig. S8 β-diversity of bacterial communities at coral microbiome transplantation experiment start. Differences between the microbiomes of donor coral fragments, inocula, and recipient corals at experiment start are highlighted using non-metric Multidimensional Scaling (nMDS) and Bray-Curtis dissimilarities. Group differences based on dissimilarities and dispersion were tested using PERMANOVA and BETADISPER analysis and p and F values are reported (R package *vegan*). (**A**) *Pocillopora* sp. and (**B**) *Porites* sp. experiment.

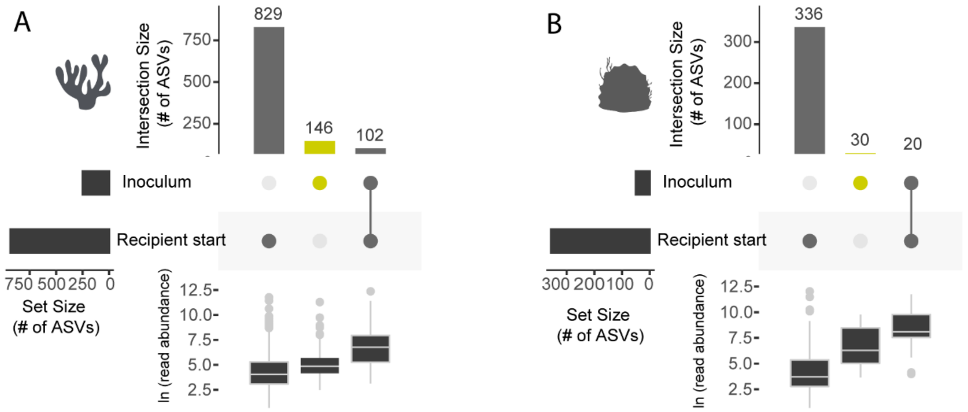

Fig. S9 Unique donor bacteria contained in the inoculum. Intersections of the inoculum with the set of recipient corals at the start of the experiments were performed in *UpsetR* to determine amplicon sequence variants (ASVs), exclusively unique for the inoculum (marked in yellow). Set sizes are indicated by horizontal bars, unique and intersection group sizes are indicated by vertical bars. Group sizes are indicated by horizontal bars, vertical bars indicate # of ASVs unique or shared as denoted by dots and connected dots below. A boxplot depicts the ln-transformed read count per set, as an indicator for their abundance. (**A**) *Pocillopora* (**B**) *Porites* experiment.

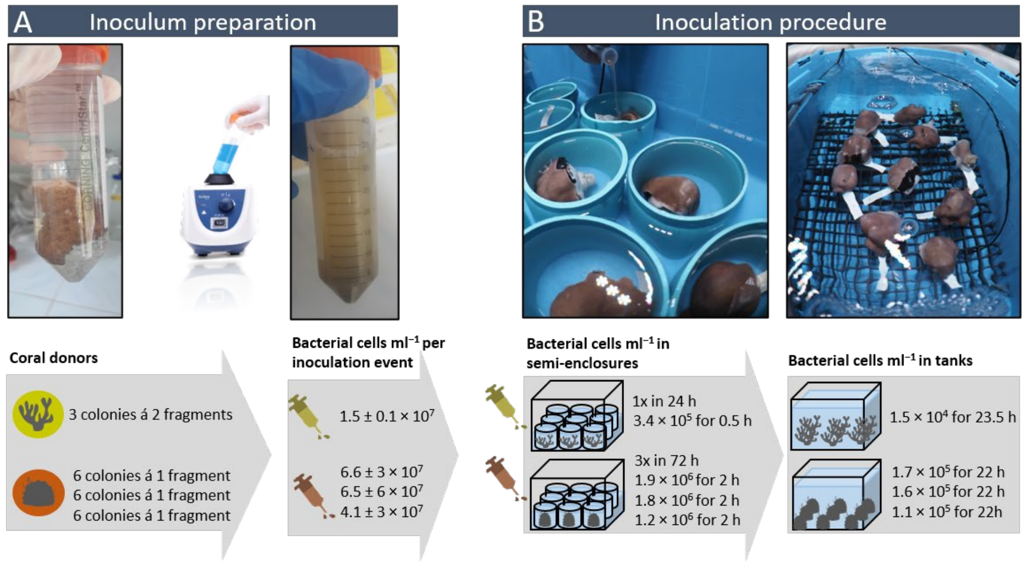

Fig. S10 Bacterial enrichment applied for the coral microbiome transplantation experiments. (**A**) Inocula were produced by homogenization of tissues from donor coral fragments by adopting protocols previously employed for the transmission of coral diseases (photos show homogenization of tissue from a *Pocillopora* fragment). Below the bacterial cell densities of the inoculum pools are shown. (**B**) Recipient fragments were inoculated inside semi-enclosures made of PVC tubes. The tubes were removed after the amount of time indicated below and water volume in the experimental tanks were increased, while decreasing bacterial cell densities as indicated below (photos show *Porites* fragments). Bacterial cell densities (cells ml^{-1}) and incubation times within the inoculation treatments are shown. Inoculation of *Pocillopora* was performed once. Inoculation of *Porites* was performed three times over the course of three days.

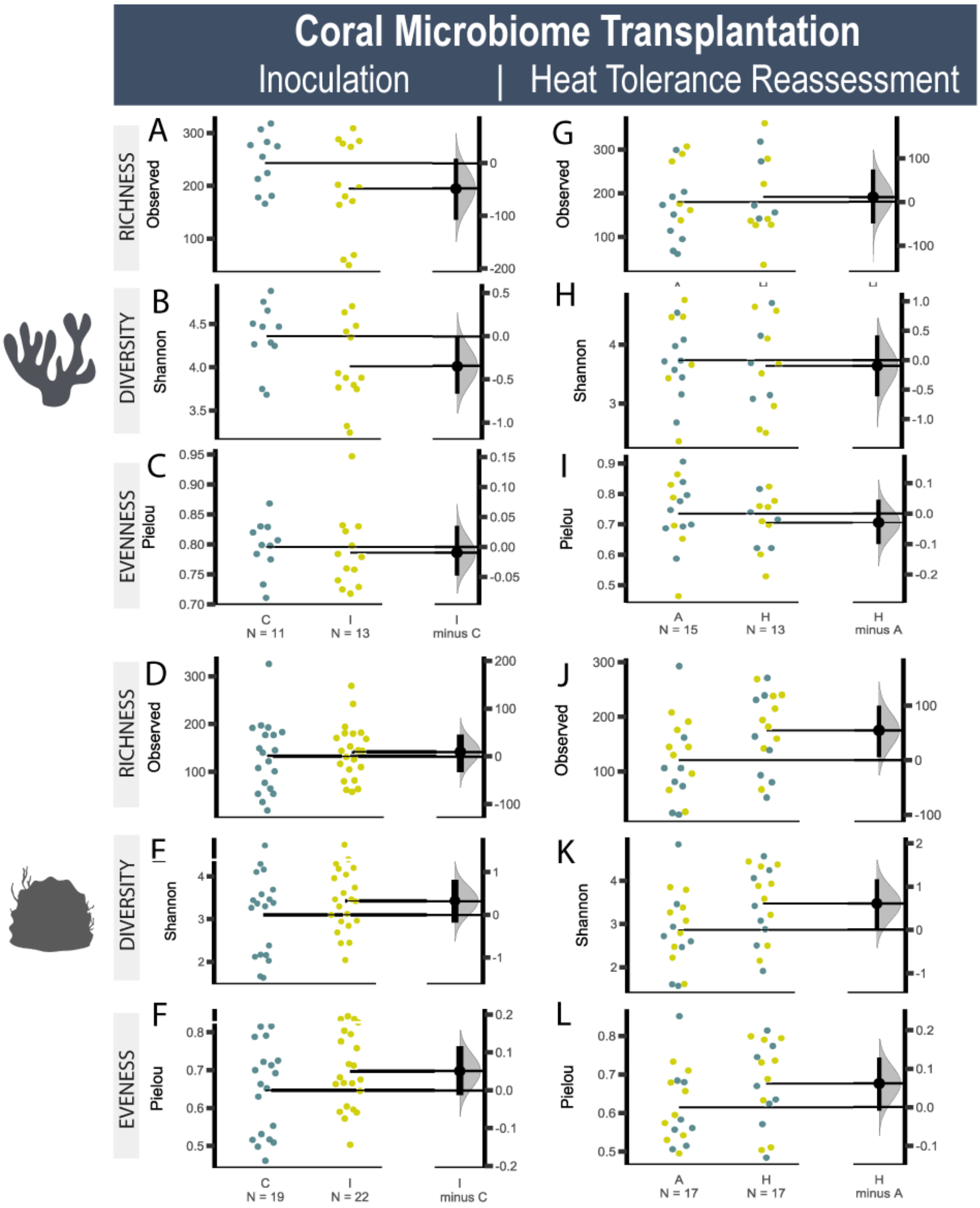

Fig. S11 Effects of inoculation treatment and heat exposure on the α-diversity metrics of coral recipient microbiomes. α-diversity metrics (i.e., Observed ASV richness, Shannon diversity index, and Pielou's evenness) are compared between the treatment groups 'I' recipients and 'C' FSW control group. Comparisons (**A-C**) for *Pocillopora* and (**D-F**) for *Porites*. Comparison between treatments '29 °C' and '34 °C' are presented in (**G-I**) for *Pocillopora* and (**J-L**) for *Porites*. Recipients are color coded: yellow = 'I' recipients, teal = 'C' sterile-filtered seawater (FSW) control group. Raw data are shown using swarm plots. Effect sizes are presented using Cumming estimation plots and depict mean difference between the treatment groups using Cohen's d and a 95% confidence interval (CI). CIs are indicated by vertical error bars. 'A' = ambient treatment of 29 °C; 'H' = heat exposure of 34 °C.

Supplementary Tables

Table S1 Generalized linear/ linear mixed effect models for coral response variables. Site = site of origin ('HighVar' = high variability site, 'LowVar' = low variability site); Temp = Temperature treatment ('34 °C' and '29 °C'); HT = heat tolerance; Inoc = Coral microbiome transplantation inoculation treatment ('I' recipients and 'C' FSW control group); Significant *p*-values are displayed in **bold**.

Experimental part (model)	Fixed effects	Estimate	Std. error	*t* value	Pr (<|z|)
***Pocillopora* sp.**					
**Δ-bleaching score**					
**HT assessment (lme)**	**Site**	0.022	0.341	0.065	0.947
	**Temp**	-0.922	0.332	-2.775	**0.011**
	**Site x Temp**	0.422	0.476	0.887	0.385
**Inoculation (glmer)**	**Inoc**	-0.0002	0.016	-0.012	0.991
**HT reassesement (lme)**	**Inoc**	-0.0001	0.036	0.000	1.000
	**Temp**	-1.700	0.036	-4.761	**<0.001**
	**Inoc x Temp**	1.100	0.051	2.178	**0.045**
**Δ-effective quantum yield**					
**HT assessment (glmer)**	**Site**	-0.001	0.006	-0.084	0.933
	**Temp**	-0.099	0.004	-2.612	**0.009**
	**Site x Temp**	0.010	0.005	1.858	0.063
**Inoculation (lme)**	**Inoc**	0.003	0.005	0.592	0.564
**HT reassesement (glmer)**	**Inoc**	-0.0001	0.002	-0.061	0.951
	**Temp**	-0.004	0.002	-1.980	**0.048**
	**Inoc x Temp**	-0.003	0.003	-0.981	0.327
***Porites* sp.**					
**Δ-bleaching score**					
**HT assessment (glmer)**	**Site**	-0.087	0.031	-2.769	**0.006**
	**Temp**	-0.126	0.016	-7.687	**<0.001**
	**Site x Temp**	0.116	0.024	4.786	**<0.001**
**Inoculation (glmer)**	**Inoc**	0.005	0.009	0.541	0.588
**HT reassessment (glmer)**	**Inoc**	-0.021	0.019	-1.096	0.273
	**Temp**	-0.156	0.019	-8.289	**<0.001**
	**Inoc x Temp**	0.093	0.027	3.479	**<0.001**
**Δ-effective quantum yield**					
**HT assessment (glmer)**	**Site**	0.007	0.002	4.639	**<0.001**
	**Temp**	0.003	0.001	2.307	**0.021**
	**Site x Temp**	0.0006	0.002	-0.302	0.763
**Inoculation (glmer)**	**Inoc**	0.0005	0.0003	1.692	0.091
**HT reassessment (glmer)**	**Inoc**	0.002	0.001	2.728	**0.006**
	**Temp**	-0.002	0.001	-3.011	**0.003**
	**Inoc x Temp**	-0.002	0.001	-2.255	**0.024**

Table S2 Post hoc test results for coral response variables 'HighVar' = high variability site; 'LowVar' = low variability site; HT = heat tolerance; '34 °C' = heat treatment; '29 °C' = ambient treatment; 'I' = recipients of inoculation; 'C' = FSW control group. Significant *p*-values results are displayed in **bold**.

**Experimental part**	**Contrast**	estimate	SE	Z ratio	*p*-value
	***Pocillopora* sp.**				
	**Δ-bleaching score**				
**HT assessement**	**LowVar x 29 °C | LowVar x 34 °C**	0.922	0.332	2.775	0.051
	**HighVar x 29 °C | HighVar x 34°C**	0.500	0.341	1.466	0.474
	**LowVar x 34 °C | HighVar x 34°C**	-0.444	0.341	-1.303	0.571
**HT reassessment**	**C x 29 °C | C x 34 °C**	1.700	0.357	4.761	**0.002**
	**I x 29 °C | I x 34 °C**	0.600	0.357	1.680	0.375
	**C x 34 °C | I x 34 °C**	-1.100	0.357	-3.081	**0.041**
	**Δ-effective quantum yield**				
**HT assessement**	**LowVar x 29 °C | LowVar x 34 °C**	0.010	0.004	2.512	**0.045**
	**HighVar x 29 °C | HighVar x 34°C**	-0.000	0.004	-0.026	1.000
	**LowVar x 34 °C | HighVar x 34°C**	-0.009	0.006	-1.686	0.331
**HT reassessment**	**C x 29 °C | C x 34 °C**	0.004	0.002	1.980	0.1953
	**I x 29 °C | I x 34 °C**	0.006	0.002	3.365	**0.004**
	**C x 34 °C | I x 34 °C**	0.003	0.002	1.447	0.470
	***Porites* sp.**				
	**Δ-bleaching score**				
**HT assessment**	**LowVar x 29 °C | LowVar x 34 °C**	0.126	0.016	7.687	**<0.001**
	**HighVar x 29 °C | HighVar x 34°C**	0.011	0.018	0.596	0.933
	**LowVar x 34 °C | HighVar x 34°C**	-0.029	0.032	-0.909	0.800
**HT reassessment**	**C x 29 °C | C x 34 °C**	0.156	0.019	8.289	**<0.001**
	**I x 29 °C | I x 34 °C**	0.064	0.019	3.370	**0.004**
	**C x 34 °C | I x 34 °C**	-0.072	0.019	-3.823	**0.001**
	**Δ-effective quantum yield**				
**HT assessment**	**LowVar x 29 °C | LowVar x 34 °C**	-0.003	0.014	-2.307	0.096
	**HighVar x 29 °C | HighVar x 34°C**	-0.003	0.015	-1.723	0.312
	**LowVar x 34 °C | HighVar x 34°C**	-0.007	0.015	-4.240	**0.001**
**HT reassessment**	**C x 29 °C | C x 34 °C**	0.002	0.001	3.011	**0.014**
	**I x 29 °C | I x 34 °C**	0.004	0.001	6.183	**<0.001**
	**C x 34 °C | I x 34 °C**	0.0003	0.001	0.458	0.968

Table S3. Generalized linear/ linear mixed effect models and post hoc tests for α-diversity metrics (Observed richness, Shannon diversity, and Pielou's eveness). Metrics were analysed within each sampling timepoint (start, inoculation, and heat tolerance (HT) reassessment) for (**A**) *Pocillopora* and (**B**) *Porites*. At 'start' differences between the groups, donor, recipient, and inoculum, were assessed. After inoculation the effect of coral microbiome transplantation inoculation (Inoc) was evaluated for the groups 'I' recipients and 'C' FSW control group, as well as after the heat exposure the effect of temperatures (Temp), '34 °C' and '29 °C, on the two groups was tested. (**C**) Significant post hoc tests. Significant *p*-values results are displayed in **bold**.

**(A)**		**Effects**	**SumSq**	**MeanSq**	**t-value**	**Pr (<|z|)**
**Start**	**Observed**	**Start group**	1384.371	692.186	0.955	0.402
	**Shannon**	**Start group**	0.711	0.356	0.951	0.403
	**Pielou**	**Start group**	0.028	0.014	0.801	0.463
		**Fixed Effects**	**Estimate**	**Std. error**	**t-value**	**Pr (<|z|)**
**Inoculation**	**Observed**	**Inoc**	-0.225	0.163	-1.377	0.169
	**Shannon**	**Inoc**	-0.316	0.151	-2.090	0.051
	**Pielou**	**Inoc**	-0.010	0.022	-0.434	0.669
**HT reassessment**	**Observed**	**Inoc**	70.363	41.594	1.692	0.106
		**Temp**	67.007	45.132	1.485	0.153
		**Inoc x Temp**	-111.530	61.340	-1.818	0.084
	**Shannon**	**Inoc**	0.183	0.371	0.494	0.627
		**Temp**	0.056	0.400	0.139	0.891
		**Inoc x Temp**	-0.348	0.546	-0.637	0.531
	**Pielou**	**Inoc**	-0.033	0.046	-0.733	0.472
		**Temp**	-0.065	0.050	-1.298	0.209
		**Inoc x Temp**	0.057	0.067	0.843	0.409

**(B)**		**Effects**			**Chi-2**	**Pr (<|z|)**
**Start**	**Observed**	**Start group**			5.222	0.073
	**Shannon**	**Start group**			3.854	0.146
		**Effects**	**SumSq**	**MeanSq**	**t-value**	**Pr (<|z|)**
	**Pielou**	**Start group**	0.044	0.022	4.672	**0.020**
		**Fixed Effects**	**Estimate**	**Std. error**	**t-value**	**Pr (<|z|)**
**Inoculation**	**Observed**	**Inoc**	7.764	18.563	0.418	0.679
	**Shannon**	**Inoc**	0.294	0.205	1.435	0.162
	**Pielou**	**Inoc**	0.047	0.026	1.777	0.086
**HT reassessment**	**Observed**	**Inoc**	16.915	29.483	0.574	0.572
		**Temp**	45.936	31.051	1.479	0.154
		**Inoc x Temp**	14.351	41.736	0.344	0.734
	**Shannon**	**Inoc**	0.087	0.348	0.251	0.804
		**Temp**	0.505	0.367	1.377	0.183
		**Inoc x Temp**	0.179	0.492	0.364	0.719
	**Pielou**	**Inoc**	-0.015	0.042	-0.353	0.728
		**Temp**	0.039	0.044	0.899	0.379
		**Inoc x Temp**	0.038	0.059	0.638	0.530

**(C)**		**Contrast**	Diff	Lwr	upr	*p*-value
**start**	**Pielou**	**inoculum | donor**	0.044	-0.066	0.155	0.578
		**recipient | donor**	-0.069	-0.141	0.002	0.058
		**recipient | inoculum**	-0.114	-0.225	-0,002	**0.045**

Table S4 PERMANOVA analyses and pairwise tests. (**A**) Differences based on Bray-Curtis dissimilarities were tested by PERMANOVA with 9,999 permutations. Results are shown per experiment (*Pocillopora*, *Porites*) and experimental part (start = start experiment, Inoculation = end inoculation phase, HT reassessment = end of heat tolerance reassessment). Applied factors: sample type = sample types of all experiments ('*Pocillopora*', '*Porites*', 'seawater source tank', and 'seawater experimental tank'), start group = start sample types ('donor', 'inoculum', and 'recipient'), Inoc = Coral microbiome transplantation inoculation treatment ('I' recipients and 'C' FSW control group), Temp = Temperature treatment ('34 °C' and '29 °C'). (**B**) Pairwise tests were conducted when homogeneity of variance was given. Significant *p*-values are displayed in **bold**.

(A)	Experimental part	Factor	Df	Sum of Sqs	R^2	F	Pr (>F)
	**Both experiments**	**Sample type**	3	21.341	0.268	25.133	**<0.001**
Pocillopora	**Start**	**Start group**	2	1.875	0.289	4.073	**<0.001**
	**Inoculation**	**Inoc**	1	0.3512	0.077	1.827	**0.013**
	**HT reassessment**	**Inoc x Temp**	3	1.356	0.195	1.944	**<0.001**
Porites	**Start**	**Start group**	2	4.161	0.650	21.339	**<0.001**
	**Inoculation**	**Inoc**	1	0.328	0.077	0.0348	0.179
	**HT reassessment**	**Inoc x Temp**	3	0.9924	0.122	1.389	0.132

(B)	Experimental part	Contrast	Donor	Inoculum	C x 29 °C	C x 34 °C	I x 29 °C
Pocillopora	**HT reassessment**	**C x 34 °C**			0.29	NA	NA
		**I x 29 °C**			**0.021**	**0.021**	NA
		**I x 34 °C**			**0.036**	**0.021**	**0.006**
Porites	**Start**	**Inoculum**	0.323	NA			
		**Recipient**	**0.003**	**0.006**			
	**HT reassessment**	**C x 34 °C**			0.222	NA	NA
		**I x 29 °C**			0.845	0.228	NA
		**I x 34 °C**			0.222	0.832	0.222

Table S5 BETADISPER analyses and pairwise tests. (**A**) Homogeneity of variances based on Bray-Curtis dissimilarities were tested by BETADISPER. Results are shown per experiment (*Pocillopora*, *Porites*) and experimental part (start = start experiment, Inoculation = end inoculation phase, HT reassessment = end of heat tolerance reassessment). Applied groups: sample type = sample types of all experiments ('*Pocillopora*', '*Porites*', 'seawater source tank', and 'seawater experimental tank'), start group = start sample types ('donor', 'inoculum', and 'recipient'), Inoc = Coral microbiome transplantation inoculation treatment ('I' recipients and 'C' FSW control group), Temp = Temperature treatment ('34 °C' and '29 °C'). (**B**) Pairwise tests were conducted if homogeneity of variance was not given. Significant *p*-values are displayed in **bold**.

(A)	Experimental part	Groups	Df	Sum of Sqs	Mean Sq	F	Pr (>F)
	**Both experiments**	**Sample type**	3	0.678	0.227	9.586	**<0.001**
Pocillopora	**Start**	**Start group**	3	0.323	0.162	9.044	**<0.001**
	**Inoculation**	**Inoc**	1	0.036	0.036	2.910	0.1
	**HT reassessment**	**Inoc x Temp**	3	0.045	0.015	1.970	0.172
Porites	**Start**	**Start group**	2	0.231	0.115	2.520	0.092
	**Inoculation**	**Inoc**	1	0.002	0.002	0.087	0.776
	**HT reassessment**	**Inoc x Temp**	3	0.029	0.01	0.162	0.919

(B)	Experimental part	Contrast	Diff	Lwr	Upr	*p*
	**Both experiments**	***Porites* | *Pocillopora***	-0.021	-0.082	0.040	0.813
		**Seawater source tank | *Pocillopora***	-0.276	-0.416	-0.135	**<0.001**
		**Seawater experimental tank | *Pocillopora***	-0.08	-0.170	0.013	0.122
		**Seawater source tank | *Porites***	-0.255	-0.393	-0.117	**<0.001**
		**Seawater experimental tank | *Porites***	-0.058	-0.147	0.031	0.333
		**Seawater experimental tank | Seawater source tank**	0.198	0.042	0.352	**0.006**
Pocilliopora	**Start**	**Inoculum | donor**	-0.361	-0.600	-0.122	**0.002**
		**Recipient | donor**	-0.071	-0.262	0.121	0.621
		**Recipient | inoculum**	0.291	0.110	0.480	**0.002**

Table S6 Summary of tank conditions during initial heat tolerance (HT) assessment. Temperature, oxygen, light intensity and salinity data (mean ± SD) are presented for the duration of the HT assay for both coral species. Temperature is specifically summarized for the temperature-peak period for each treatment (i.e., '29 °C' and '34 °C'). Two HT assays for *Porites* corals were run separately, as *Porites* corals from each respective high and low variability habitat required different light regimes. Sites of origin: 'HighVar' = high variability site; 'LowVar' = low variability site.

**Corals 'Site of origin'**	**Treatments**	Temperature (°C)	Peak temperature (°C)	Dissolved oxygen (mg/L)	Light intensity (µmol m^{-2} s^{-2})	Salinity (PSU)
Pocillopora **'HighVar' | 'LowVar'**	***'29 °C'***	29.82 ± 0.35	30.08 ± 0.19	7.61 ± 0.02	74 ± 3	32.7 ± 0.1
	'34 °C'	30.99 ± 2.03	33.89 ± 0.56	7.63 ± 0.01	75 ± 5	32.4 ± 0.1
Porites **'HighVar'**	***'29 °C'***	29.26 ± 0.29	29.44 ± 0.10	8.50 ± 0.22	591 ± 95	NA
	'34 °C'	30.37 ± 1.98	34.52 ± 0.06	8.17 ± 0.29	612 ± 77	NA
Porites **'LowVar'**	***'29 °C'***	29.14 ± 0.18	29.35 ± 0.18	8.20 ± 0.27	83 ± 8	NA
	'34 °C'	30.46 ± 2.01	34.27 ± 0.16	8.16 ± 0.29	84 ± 9	NA

Table S7 Summary of tank conditions during coral microbiome transplantation (CMT) experiments. Temperature, oxygen, light intensity, and salinity data (mean ± SD) are presented for the duration of the entire CMT procedure and heat tolerance (HT) reassessment. Temperature is also specifically summarized for the time of inoculation ('I' recipients and 'C' FSW control group) and during the temperature-peak for the two treatments ('29 °C' and '34 °C').

**Corals**	**Treatments**	Temperature (°C)	CMT inoculation temperature (°C)	HT assay peak temperature (°C)	Dissolved oxygen (mg/L)	Light intensity (µmol m^{-2} s^{-2})	Salinity (PSU)
Pocillopora	***C x 29 °C***	29.42 ± 0.35	29.46 ± 0.46	29.52 ± 0.15	7.41 ± 0.01	74 ± 8	32.9
	I x 29 °C	29.44 ± 0.32	29.45 ± 0.35	29.64 ± 0.23	7.43 ± 0.01	83 ± 11	32.9
	C x 34 °C	30.46 ± 1.89	29.45 ± 0.40	34.37 ± 0.07	7.42 ± 0.01	68 ± 6	32.8
	I x 34 °C	30.41 ± 1.84	29.44 ± 0.39	34.24 ± 0.05	7.42 ± 0.01	74 ± 9	32.9
Porites	***C x 29 °C***	29.07 ± 0.15	28.99 ± 0.14	29.15 ± 0.16	8.18 ± 0.08	87 ± 9	NA
	I x 29 °C	29.08 ± 0.37	28.96 ± 0.50	29.18 ± 0.11	8.13 ± 0.09	89 ± 8	NA
	C x 34 °C	29.86 ± 1.57	29.21 ± 0.24	34.32 ± 0.06	8.15 ± 0.06	87 ± 11	NA
	I x 34 °C	29.91 ± 1.59	29.26 ± 0.20	34.38 ± 0.07	8.13 ± 0.05	85 ± 11	NA

Supplementary Datasets

Legends for datasets (Excel Files)

Dataset S1 Analysis table of coral response variables. 'LowVar' site = Racha Island east shore, 'HighVar' site (*Pocillopora*) = Racha Island west shore, 'HighVar' site (*Porites*) = Panwa reef flat; heat tolerance (HT) assessment treatments: '34 °C' and '29 °C'; Inoculation treatments: 'I' = inoculation; 'C' = sterile-filtered seawater (FSW) control group; n = replicate fragment numbers; mean difference = mean difference between start and end of a treatment; SD = standard deviation; SE = standard error; CI = confidence interval.

Dataset S2 Analysis tables of microbiome data. Count tables are provided for (**A**) 'filt-10' data and (**B**) rarefied data (subsampled to 4 000 reads). Tables enclose read abundance counts per amplicon sequence variant (ASV), experiment metadata (i.e., treatment groups), and SILVA classification. TYPE = sample type; POC = *Pocillopora*; POR = *Porites*; SW = seawater. Timepoints include: t1 = start, t2 = end of inoculation, t3 = timepoint of water collection from the source tank during heat tolerance (HT) reassessment, t4 = end of HT reassessment. I = inoculation; C = FSW control group; H = heat exposure treatment 34 °C; A = ambient temperature treatment 29 °C.

Dataset S3 Full table of potentially transmitted bacteria in the (A) *Pocillopora* and (B) *Porites* experiment. Amplicon sequence variants (ASVs) exclusively shared between the inoculum and the 'I' recipient' group after inoculation are provided each with their respective SILVA based taxonomy and sequence. Read abundances for each potentially transmitted ASV show its occurrence within the different treatment groups. ASVs are marked in 'green', when also detected in the donor samples. Those, also detected in seawater samples, are marked in 'blue'. ASVs are marked in red, when detected in the 'I' recipient group at the end of heat tolerance reassessment (i.e., ASVs that persisted within the recipients' microbiomes until the very end of the experiment).

Dataset S4 Quality control samples and clean-up of microbiome data. Tables show host-origin and contaminant amplicon sequence variant (ASV) sequences that were removed from the microbiome data set prior to downstream analyses. (**A**) Table shows ASV sequences of host-origin as matched with GenBank (NCBI). (**B**) Table shows ASVs identified as contaminants using DNA Extraction Kit and PCR blank samples. Stacked bar charts show bacterial community compositions of (**C**) extraction kit blank samples and (**D**) PCR blank samples. Additionally, scoring tables for contaminant ASVs are shown.

Legend for datasets (Word Document File)

Dataset S5 Protocol of raw read processing using QIIME2 V2019.7.

References

1. Osborne AR, Burch TL. Internal solitons in the Andaman Sea. *Science (80-)* 1980; **208**: 451–460.
2. Vlasenko V, Stashchuk N. Three-dimensional shoaling of large-amplitude internal waves. *J Geophys Res Ocean* 2007; **112**: 1–15.
3. Wall M, Schmidt GM, Janjang P, Khokiattiwong S, Richter C. Differential Impact of Monsoon and Large Amplitude Internal Waves on Coral Reef Development in the Andaman Sea. *PLoS One* 2012; **7**: 1–10.
4. Brown B, Dunne RP, Somerfield PJ, Edwards AJ, Simons WJF, Phongsuwan N, et al. Long-term impacts of rising sea temperature and sea level on shallow water coral communities over a ~40 year period. *Sci Rep* 2019; **9**: 1–12.
5. Garren M, Azam F. New method for counting bacteria associated with coral mucus. *Appl Environ Microbiol* 2010; **76**: 6128–6133.
6. Roik A, Röthig T, Roder C, Ziegler M, Kremb SG, Voolstra CR. Year-long monitoring of physico-chemical and biological variables provide a comparative baseline of coral reef functioning in the central Red Sea. *PLoS One* 2016; **11**: 1–34.
7. Kozich JJ, Westcott SL, Baxter NT, Highlander SK, Schloss PD. Development of a dual-index sequencing strategy and curation pipeline for analyzing amplicon sequence data on the MiSeq Illumina sequencing platform. *Appl Environ Microbiol* 2013; **79**: 5112–5120.
8. Martin M. Cutadapt removes adapter sequences from high-throughput sequencing reads. *EMBnet.journal* 2011; **17**: 10.
9. Callahan BJ, McMurdie PJ, Rosen MJ, Han AW, Johnson AJA, Holmes SP. DADA2: High-resolution sample inference from Illumina amplicon data. *Nat Methods* 2016; **13**: 581–583.
10. Quast C, Pruesse E, Yilmaz P, Gerken J, Schweer T, Yarza P, et al. The SILVA ribosomal RNA gene database project: Improved data processing and web-based tools. *Nucleic Acids Res* 2013; **41**: D590–D596.
11. Pedregosa F, Varoquaux G, Gramfort A, Michel V, Thirion B, Grisel O, et al. Scikit-learn: Machine learning in Python. *J Mach Learn Res* 2011; **12**: 2825–30.
12. Bokulich NA, Kaehler BD, Rideout JR, Dillon M, Bolyen E, Knight R, et al. Optimizing taxonomic classification of marker-gene amplicon sequences with QIIME 2's q2-feature-classifier plugin. *Microbiome* 2018; **6**: 90.
