# Supplementary material for: Towards enhancing coral heat tolerance: a “microbiome transplantation” treatment using inoculations of homogenized coral tissues
Source: Microbiome. 2021 May 6;9:102. doi: 10.1186/s40168-021-01053-6 (PMC8103578; doi:10.1186/s40168-021-01053-6)
Supplement: Supplementary file 7 — Additional file 6: Dataset S5. Protocol of raw read processing using QIIME2 V2019.7. [file 40168_2021_1053_MOESM7_ESM.docx]

**Dataset S5 Protocol of raw read processing using Qiime2 V2019.7.**

**#Coral Microbiome Transplantation Experiments**

**#16S amplicon data protocol**

**#Analysis Code 2019/2020**

#------PRIMER INFO

# Primers used:

# V34 341F-806R IKMB

# Primers used: fwd CCTACGGGAGGCAGCAG rev GGACTACHVGGGTWTCTAAT 341-806 466bp + # max 2*12 = 490 bp

# primer fwd in Silva 132: 6388-6426 primer rev: 23446-25318

--p-f-primer CCTACGGGAGGCAGCAG

--p-r-primer GGACTACHVGGGTWTCTAAT

#------ QIIME2

source activate qiime2-2019.7

# 'LIB3' data

# load data:

qiime tools import \

--type 'SampleData[PairedEndSequencesWithQuality]' \

--input-path PairedEndFastqManifestLib3.csv \

--output-path paired-end-demux.qza \

--input-format PairedEndFastqManifestPhred33

# Create a visualization artifact

qiime demux summarize \

--i-data paired-end-demux.qza \

--o-visualization paired-end-demux.qzv

# 'LIB3rep' data

# load data:

qiime tools import \

--type 'SampleData[PairedEndSequencesWithQuality]' \

--input-path PairedEndFastqManifestLib3rep.csv \

--output-path paired-end-demux.qza \

--input-format PairedEndFastqManifestPhred33

# Create a visualization artifact

qiime demux summarize \

--i-data paired-end-demux.qza \

--o-visualization paired-end-demux.qzv

# clipping non-16S sequences

# clip

# CCTACGGGAGGCAGCAG

# GGACTACHVGGGTWTCTAAT

# Also clip non 16S sequences occurring in ASV with an abundance of >40 reads in Lib3

# TGGGGAATATTGGACAATGAACGA

# GAGGGGAAGAAGGGAGGCAGCA

# TCGAGAATCATTCACAATGGGGGAAACC

# CGAAAGAAGGGAGGAAGCA

# TCTACGGGAGTCAGCA

#Lib3

# clip IKMB data V34 Paired End:

qiime cutadapt trim-paired \

--i-demultiplexed-sequences paired-end-demux.qza \

--p-front-f CCTACGGGAGGCAGCAG \

--p-front-r GGACTACHVGGGTWTCTAAT \

--p-front-f TGGGGAATATTGGACAATGAACGA \

--p-front-f GAGGGGAAGAAGGGAGGCAGCA \

--p-front-f TCGAGAATCATTCACAATGGGGGAAACC \

--p-front-f CGAAAGAAGGGAGGAAGCA \

--p-front-f TCTACGGGAGTCAGCA \

--o-trimmed-sequences paired-end-demux.trim.qza \

--output-dir $PWD/trim2

#viz

qiime demux summarize \

--i-data $PWD/trim2/paired-end-demux.trim.qza \

--o-visualization paired-end-demux.trim.qzv

--output-dir $PWD/trim2

Lib3 Trim TRIMMED READS


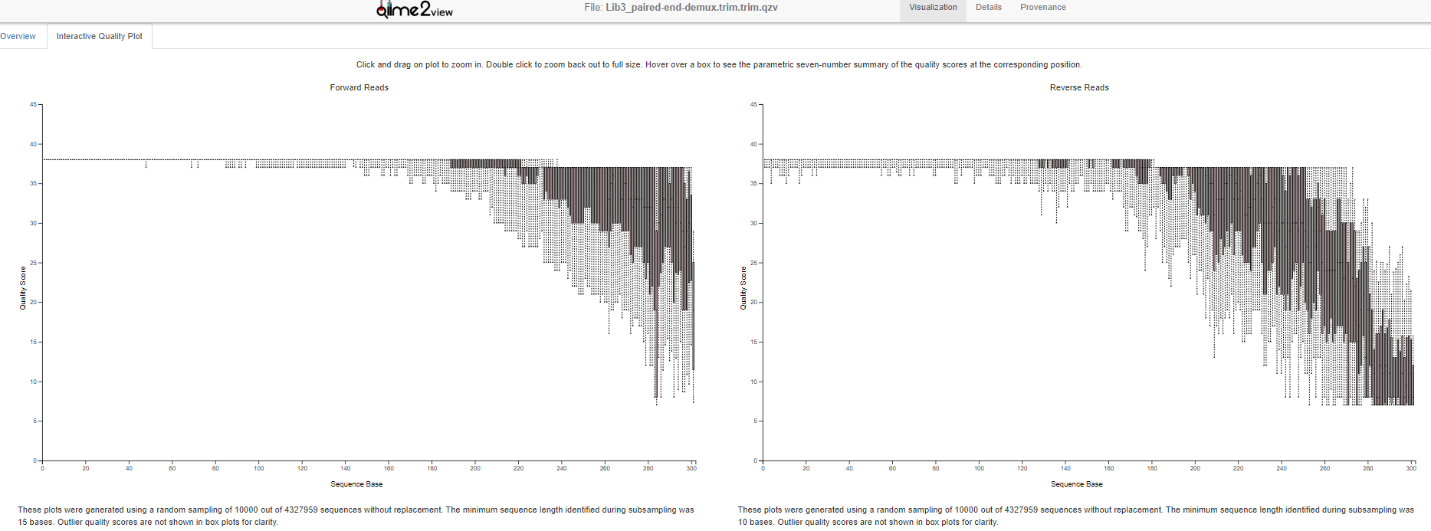


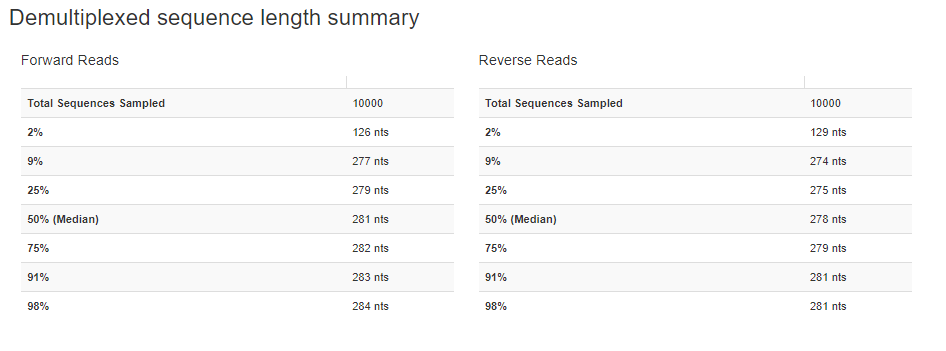


#Lib3rep

# clip IKMB data V34 Paired End:

qiime cutadapt trim-paired \

--i-demultiplexed-sequences paired-end-demux.qza \

--p-front-f CCTACGGGAGGCAGCAG \

--p-front-r GGACTACHVGGGTWTCTAAT \

--o-trimmed-sequences paired-end-demux.trim.qza

#viz

qiime demux summarize \

--i-data paired-end-demux.trim.qza \

--o-visualization paired-end-demux.trim.qzv


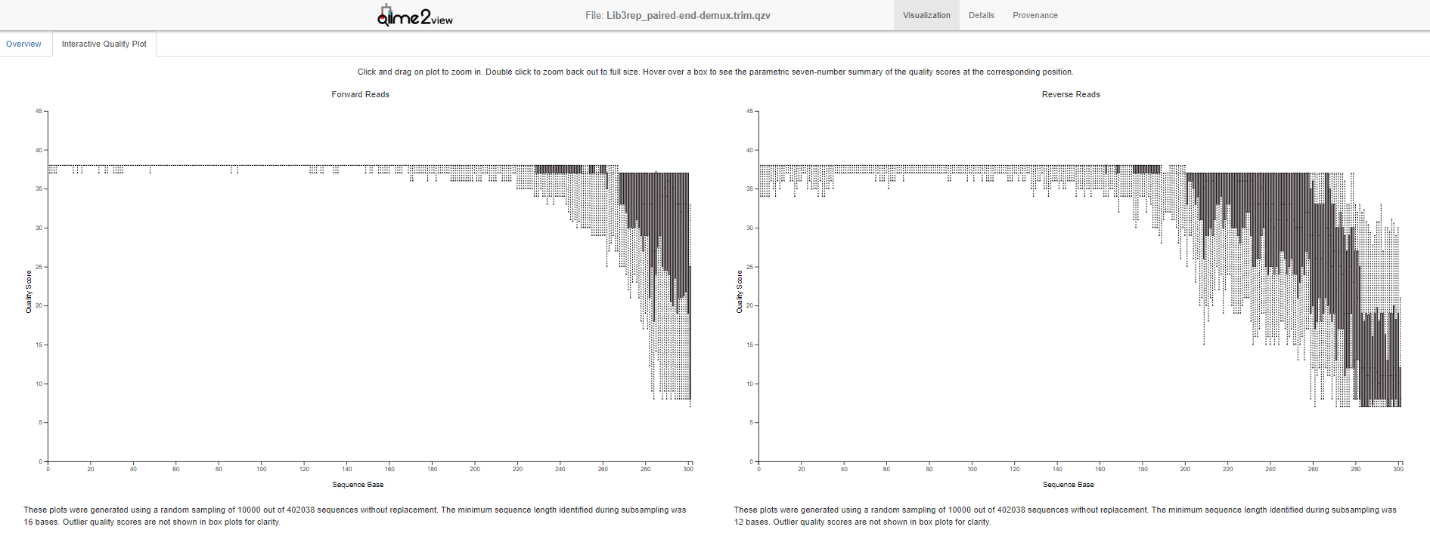


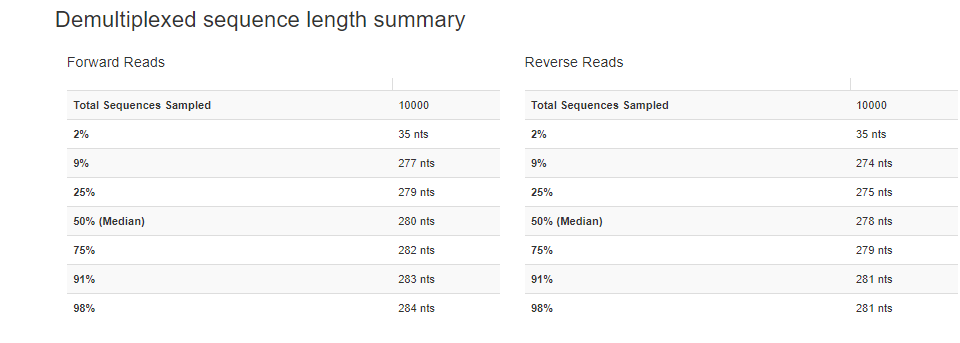


#------DENOISING, ASV GENERATION (DADA2)

# TRUNCATING sequences (overlap should be min. >20 bp)

# Total length of this 16S V34 region = 341F-806R = 466bp

# Lib3

# Fo reads @277 bp

# Re reads @220 bp

# Makes an overlap of 31 bp

#Lib3rep

# Fo reads @278 bp

# Re reads @230 bp

# Makes an overlap of 42 bp

#Lib3

# Paired End: DADA2 denoising

qiime dada2 denoise-paired \

--i-demultiplexed-seqs paired-end-demux.trim.qza \

--p-trunc-len-f 277 \

--p-trunc-len-r 220 \

--o-representative-sequences Lib3-paired-rep-seqs-dada2.qza \

--o-table Lib3-paired-table-dada2.qza \

--o-denoising-stats Lib3-paired-dada2-stats.qza \

--p-n-threads 8 \

--verbose

#viz

qiime feature-table tabulate-seqs \
 --i-data Lib3-paired-rep-seqs-dada2.qza \
 --o-visualization Lib3-paired-rep-seqs-dada2.qzv

#viz

qiime metadata tabulate \

--m-input-file Lib3-paired-dada2-stats.qza \

--o-visualization Lib3-paired-dada2-stats.qzv

qiime feature-table summarize \

--i-table Lib3-paired-table-dada2.qza \

--o-visualization Lib3-paired-table-dada2.qzv \

--m-sample-metadata-file metadata.file.Lib3.txt

| 69 % remaining sequences |
| --- |
| 3015175 sequences remain |

10330 Features in total


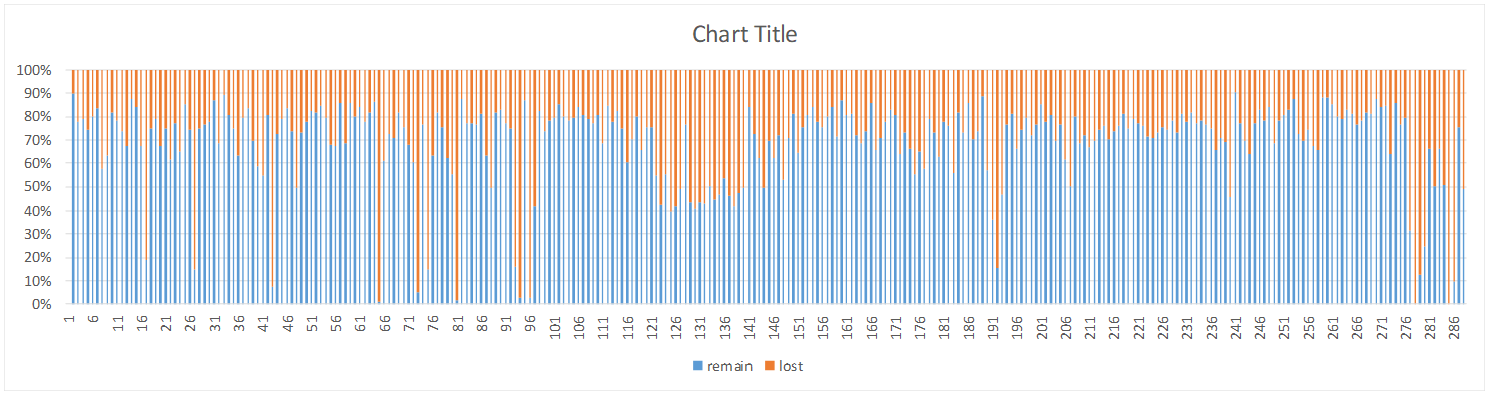


#Lib3rep

# Paired End: DADA2 denoising

qiime dada2 denoise-paired \

--i-demultiplexed-seqs paired-end-demux.trim.qza \

--p-trunc-len-f 278 \

--p-trunc-len-r 230 \

--o-representative-sequences Lib3rep-paired-rep-seqs-dada2.qza \

--o-table Lib3rep-paired-table-dada2.qza \

--o-denoising-stats Lib3rep-paired-dada2-stats.qza \

--p-n-threads 8 \

--verbose

# viz

qiime feature-table tabulate-seqs \
--i-data Lib3rep-paired-rep-seqs-dada2.qza \
--o-visualization Lib3rep-paired-rep-seqs-dada2.qzv

# viz

qiime metadata tabulate \

--m-input-file Lib3rep-paired-dada2-stats.qza \

--o-visualization Lib3rep-paired-dada2-stats.qzv

qiime feature-table summarize \

--i-table Lib3rep-paired-table-dada2.qza \

--o-visualization Lib3rep-paired-table-dada2.qzv \

--m-sample-metadata-file metadata.file.Lib3rep.txt

| 74 % remaining sequences |
| --- |
| 299620 sequences remain |

2693 Features in total


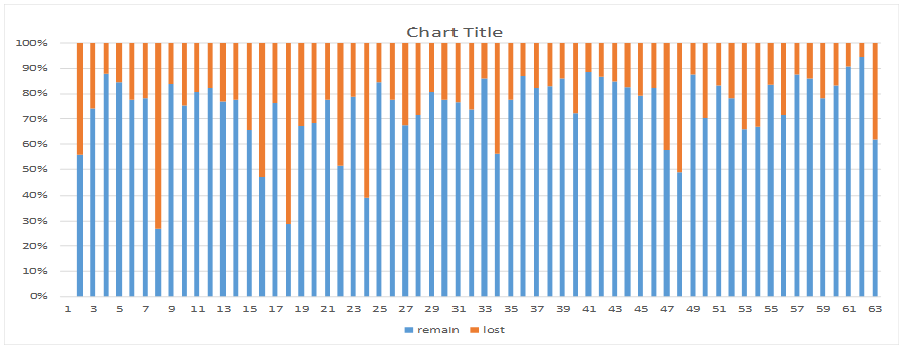


#------ MERGING DENOISED DATA SETS

qiime feature-table merge \
 --i-tables $PWD/lib3rep_processing/Lib3rep-paired-table-dada2.qza \
 --i-tables $PWD/lib3_processing/Lib3-paired-table-dada2.qza \
 --o-merged-table merge-table-dada2.qza
 qiime feature-table merge-seqs \
 --i-data $PWD/lib3rep_processing/Lib3rep-paired-rep-seqs-dada2.qza \
 --i-data $PWD/lib3_processing/Lib3-paired-rep-seqs-dada2.qza \
 --o-merged-data merge-rep-seqs.qza

#viz

qiime feature-table tabulate-seqs \
 --i-data merge-rep-seqs.qza \
 --o-visualization merge-rep-seqs.qzv

qiime feature-table summarize \
 --i-table merge-table-dada2.qza \
 --o-visualization merge-table-dada2.qzv \
 --m-sample-metadata-file metadata.file.Lib3merge.txt

| 3314795 sequences |  |  |
| --- | --- | --- |

11286 merged Features in total (Lib3 + Lib3rep = 13023 Features altogether)

#------ TRAIN CLASSIFIER (Naive Bayes, SILVA 132)

qiime tools import \

--input-path $PWD/SILVA_132_QIIME_release/rep_set/rep_set_16S_only/99/silva_132_99_16S.fna \

--output-path silva_132_99_16S_fasta.qza \

--type 'FeatureData[Sequence]'

qiime tools import \

--input-path taxonomy_7_levels.txt \

--output-path silva132_taxonomy_7_levels.qza \

--input-format HeaderlessTSVTaxonomyFormat \

--type 'FeatureData[Taxonomy]'

# Extract reference reads

# this step ca take 14h or more

qiime feature-classifier extract-reads \

--i-sequences silva_132_99_16S_fasta.qza \
--p-f-primer CCTACGGGAGGCAGCAG \
--p-r-primer GGACTACHVGGGTWTCTAAT \
--p-trunc-len 466 \

--o-reads silva132V3V4_99_16S-ref-seqs.qza

# Train the classifier

qiime feature-classifier fit-classifier-naive-bayes \
 --i-reference-reads silva132V3V4_99_16S-ref-seqs.qza \
 --i-reference-taxonomy silva132_taxonomy_7_levels.qza \
 --o-classifier Silva132V3V4-0.21.2-7level-noncons-classifier.qza

#------ CLASSIFY (sklearn)

# classify using sklearn

qiime feature-classifier classify-sklearn \

--i-classifier /media/5c679734-9376-4617-815c-d4bd4177b8b2/anna/classifier_qiime2/Silva132V3V4-0.21.2-7level-noncons-classifier.qza \

--p-confidence 0.8 \

--p-n-jobs 2 \

--p-reads-per-batch 1000 \

--i-reads merge-rep-seqs.qza \

--o-classification merge-tax-silva132_7levnoncons.qza

# viz

# check How many unassigned ?

qiime metadata tabulate \

--m-input-file $PWD/class-7level-noncons/merge-tax-silva132_7levnoncons.qza \

--o-visualization merge-tax-silva132_7levnoncons.qzv

# Barplots

qiime taxa barplot \

--i-table merge-table-dada2.qza \

--i-taxonomy $PWD/class-7level-noncons/merge-tax-silva132_7levnoncons.qza \

--m-metadata-file metadata.file.Lib3merge.3oct.txt \

--o-visualization merge-tax-silva132_7levnoncons-barplot.qzv


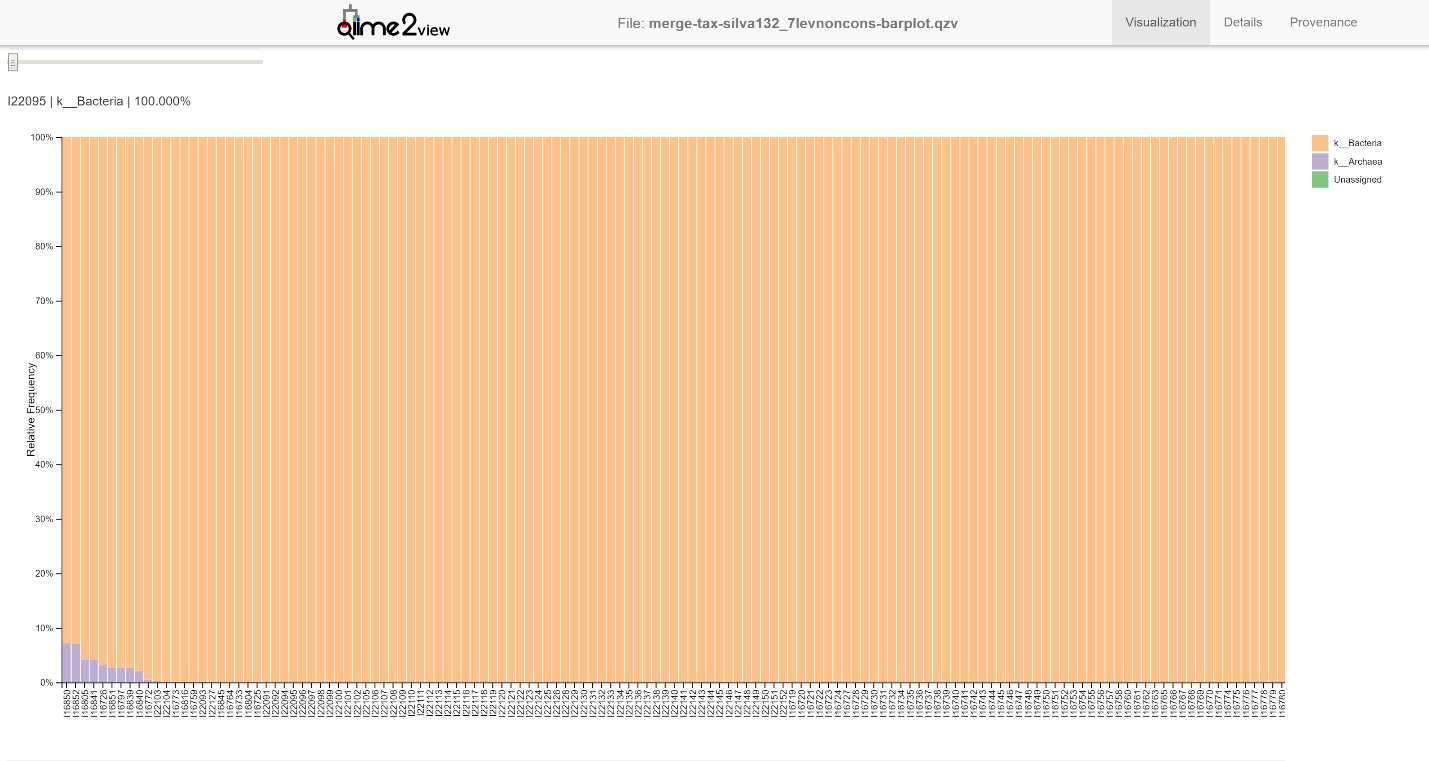


#------ FILTERING of unrelated sequences (Chloroplast, Mitochondrial, Archaeal, Unassigned sequences)

# filt chloroplasts

qiime feature-table filter-features \

--i-table merge-table-dada2.qza \

--m-metadata-file $PWD/class-7level-noncons/merge-tax-silva132_7levnoncons.qza \

--p-where "Taxon NOT LIKE '%Chloroplast%'" \

--o-filtered-table merge-table-dada2-filt-chloro.qza

# filt mitochondria

qiime feature-table filter-features \

--i-table merge-table-dada2-filt-chloro.qza \

--m-metadata-file $PWD/class-7level-noncons/merge-tax-silva132_7levnoncons.qza \

--p-where "Taxon NOT LIKE '%Mitochondria%'" \

--o-filtered-table merge-table-dada2-filt-chloromito.qza

# filt archaea

qiime feature-table filter-features \

--i-table merge-table-dada2-filt-chloromito.qza \

--m-metadata-file $PWD/class-7level-noncons/merge-tax-silva132_7levnoncons.qza \

--p-where "Taxon NOT LIKE '%%Archaea%'" \

--o-filtered-table merge-table-dada2-filt-chloromitoarch.qza

# filt unassigned

qiime feature-table filter-features \

--i-table merge-table-dada2-filt-chloromitoarch.qza \

--m-metadata-file $PWD/class-7level-noncons/merge-tax-silva132_7levnoncons.qza \

--p-where "Taxon NOT LIKE '%Unassigned%'" \

--o-filtered-table merge-table-dada2-filt-chloromitoarchunassig.qza

#VIZ filtered

# reads per samples | reads per feature

qiime feature-table summarize \

--i-table merge-table-dada2-filt-chloromitoarchunassig.qza \

--o-visualization merge-table-dada2-filt-chloromitoarchunassig.qzv \

--m-sample-metadata-file metadata.file.Lib3merge.3oct.txt

# Barplots

qiime taxa barplot \

--i-table merge-table-dada2-filt-chloromitoarchunassig.qza \

--i-taxonomy $PWD/class-7level-noncons/merge-tax-silva132_7levnoncons.qza \

--m-metadata-file metadata.file.Lib3merge.3oct.txt \

--o-visualization merge-table-dada2-filt-chloromitoarchunassig-barplot.qzv

Table summary

| Metric | Sample |
| --- | --- |
| Number of samples | 346 |
| Number of features | 10,863 |
| Total frequency | 3,000,016 |

#------ SUBSETTING experiment data

# filt INOC1+3

qiime feature-table filter-samples \

--i-table /media/5c679734-9376-4617-815c-d4bd4177b8b2/anna/lib3_qiime2/paired_v4/merge-table-dada2-filt-chloromitoarchunassig.qza \

--m-metadata-file /media/5c679734-9376-4617-815c-d4bd4177b8b2/anna/lib3_qiime2/paired_v4/metadata.file.Lib3merge.3oct.txt \

--p-where "experiment LIKE '%INOC1%'" \

--p-where "experiment LIKE '%INOC3%'" \

--o-filtered-table merge-table-dada2-filt-INOC1_3.qza

There was a problem with the command:

(1/1?) Option '--p-where' was specified multiple times in the command.

qiime feature-table filter-samples \

--i-table merge-table-dada2-filt-chloromitoarchunassig.qza \

--m-metadata-file metadata.file.Lib3merge.3oct.txt \

--p-where "experiment LIKE '%HE%'" \

--p-exclude-ids TRUE \

--o-filtered-table merge-table-dada2-filt-INOC1_3x.qza

qiime feature-table filter-samples \

--i-table merge-table-dada2-filt-INOC1_3x.qza \

--m-metadata-file metadata.file.Lib3merge.3oct.txt \

--p-where "experiment LIKE '%MOCK%'" \

--p-exclude-ids TRUE \

--o-filtered-table merge-table-dada2-filt-INOC1_3xy.qza

qiime feature-table filter-samples \

--i-table merge-table-dada2-filt-INOC1_3xy.qza \

--m-metadata-file metadata.file.Lib3merge.3oct.txt \

--p-where "experiment LIKE '%NEG%'" \

--p-exclude-ids TRUE \

--o-filtered-table merge-table-dada2-filt-INOC1_3xyz.qza

qiime feature-table filter-samples \

--i-table merge-table-dada2-filt-INOC1_3xyz.qza \

--m-metadata-file metadata.file.Lib3merge.3oct.txt \

--p-where "experiment LIKE '%emptywell%'" \

--p-exclude-ids TRUE \

--o-filtered-table merge-table-dada2-filt-INOC1_3.qza

#VIZ

# reads per samples | reads per feature

qiime feature-table summarize \

--i-table merge-table-dada2-filt-INOC1_3.qza \

--o-visualization merge-table-dada2-filt-INOC1_3.qzv \

--m-sample-metadata-file /media/5c679734-9376-4617-815c-d4bd4177b8b2/anna/lib3_qiime2/paired_v4/metadata.file.Lib3merge.3oct.txt

# Barplots

qiime taxa barplot \

--i-table merge-table-dada2-filt-INOC1_3.qza \

--i-taxonomy /media/5c679734-9376-4617-815c-d4bd4177b8b2/anna/lib3_qiime2/paired_v4/class-7level-noncons/merge-tax-silva132_7levnoncons.qza \

--m-metadata-file metadata.file.Lib3merge.3oct.txt \

--o-visualization merge-table-dada2-filt-INOC1_3-barplot.qzv

| Metric | Sample |
| --- | --- |
| Number of samples | 312 |
| Number of features | 9,593 |
| Total frequency | 2,560,337 |

#------ QUALITY CHECK rarefaction (evaluate sequencing depth)

# INOC1_3 rarefaction (ca. 10 min)

qiime diversity alpha-rarefaction \

--i-table merge-table-dada2-filt-INOC1_3.qza \

--i-phylogeny /media/5c679734-9376-4617-815c-d4bd4177b8b2/anna/lib3_qiime2/paired_v4/rooted-tree-merge-rep-seqs.qza \

--p-min-depth 100 \

--p-max-depth 15000 \

--p-steps 50 \

--p-iterations 55 \

--m-metadata-file /media/5c679734-9376-4617-815c-d4bd4177b8b2/anna/lib3_qiime2/paired_v4/metadata.file.Lib3merge.3oct.txt \

--p-metrics simpson_e \

--p-metrics simpson \

--p-metrics shannon \

--p-metrics observed_otus \

--p-metrics faith_pd \

--o-visualization merge-table-dada2-filt-INOC1_3-rarefaction.qzv


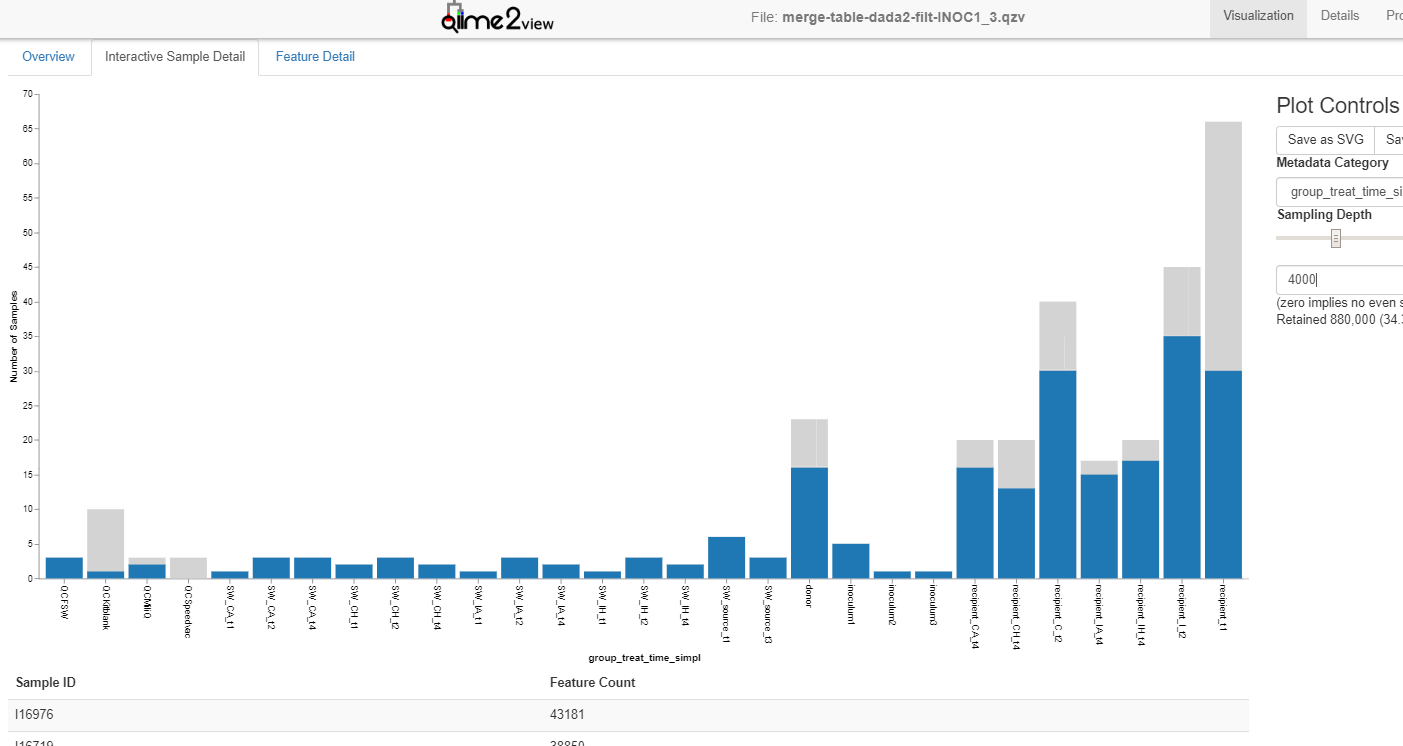


#------ EXPORT INOC1+3 experiment data and continue in --> PHYLOSEQ (R environment)

# export OTU/ASV count tables

qiime tools export \

--input-path merge-table-dada2-filt-INOC1_3.qza \

--output-path INOC1_3_phyloseq

# convert to .tsv

biom convert -i INOC1_3_phyloseq/feature-table.biom -o INOC1_3_phyloseq/asv_count.txt --to-tsv

#  now you  have an OTU table .txt

#  open it  up  in  text  edit  and  change  #OTUID  to  OTUID
